# Uncovering the Independent Role of Lipid Peroxidation in Osteoporosis Through an Integrated Bibliometric and Bioinformatics Analysis

**DOI:** 10.1155/mi/1064232

**Published:** 2026-02-16

**Authors:** Xin Li, Liqi Ng, Wei Dong, Mengen Li, Yingtao Bai, Yusong Liu, Chunbao Wu, Yu Zhou

**Affiliations:** ^1^ Department of Pharmacy, Orthopedic Hospital, Chongqing University of Chinese Medicine, Chongqing, 400012, China; ^2^ Chongqing Traditional Chinese Medicine Orthopedic Research Institute, Chongqing, 400012, China; ^3^ Institute of Orthopaedic and Musculoskeletal Science, University College London, Royal National Orthopaedic Hospital, Stanmore, London, HA7 4LP, UK, nhs.uk; ^4^ Department of Spine Surgery, Orthopedic Hospital, Chongqing University of Chinese Medicine, Chongqing, 400012, China; ^5^ Postdoctoral Research Workstation, Orthopedic Hospital, Chongqing University of Chinese Medicine, Chongqing, 400012, China

**Keywords:** autophagy, bibliometric, lipid peroxidation, osteoporosis, visualisation analysis

## Abstract

**Objective:**

This study, utilising bibliometric analysis combined with bioinformatics approaches, systematically analysed research trends in the fields of osteoporosis (OP) and autophagy (ATG) over the past two decades, with a focus on the emerging frontier of lipid peroxidation (LP). The aim was to reveal its independent role in the OP network, distinct from the ferroptosis framework.

**Methods:**

CiteSpace.6.4.R1 was utilised to perform visualisation analysis on 588 relevant articles from the Web of Science Core Collection, examining countries, institutions, authors, and keywords. Common targets between OP and key burst terms were screened via the Gene Expression Omnibus (GEO) database, followed by the construction of a protein–protein interaction (PPI) network and gene ontology (GO)/Kyoto Encyclopedia of Genes and Genomes (KEGG) enrichment analysis. Subsequently, we constructed 113 models using 12 machine learning algorithms to screen for feature genes, and the diagnostic value of key targets was validated using receiver operating characteristic (ROC) curves.

**Results:**

Bibliometric analysis indicated that the field entered a period of rapid development from 2018, with China dominating in terms of publication volume and the United States leading in academic influence. Keyword burst detection identified ‘LP’ as an emerging frontier since 2023. Bioinformatics analysis identified 127 common OP–LP targets, which are enriched in pathways such as NF‐κB, eestrogen signalling, and mitophagy. Through machine learning and MCODE module analysis, five key targets were ultimately screened: Amyloid beta precursor protein (APP), Forkhead Box O1 (FOXO1), Forkhead Box O3 (FOXO3), Jun Proto‐Oncogene (JUN), and Synuclein Alpha (SNCA). ROC curves demonstrated their good diagnostic efficacy.

**Conclusion:**

This study is the first to integrate bibliometric and bioinformatics methods, revealing the macro‐level trends in OP–ATG research and the molecular mechanisms underlying OP–LP crossover. It successfully identified five key OP–LP targets, providing a new perspective for understanding OP mechanisms and developing targeted therapies.

## 1. Introduction

Osteoporosis (OP) is a systemic metabolic bone disease characterised by reduced bone mass and deterioration of bone microarchitecture. The consequent increase in bone fragility places patients at a significantly elevated risk of fractures, making OP a significant global public health challenge [1]. The core pathological mechanism of OP lies in the imbalance of bone remodelling homeostasis, whereby osteoclast‐mediated bone resorption exceeds osteoblast‐mediated bone formation [2]. In recent years, the crucial role of autophagy (ATG), a key intracellular homeostatic regulatory mechanism, in maintaining bone metabolic balance has been progressively elucidated [3]. Moderate ATG serves to clear damaged organelles (such as dysfunctional mitochondria) and misfolded proteins, providing energy substrates for cells and alleviating oxidative stress, thereby ensuring the normal function and differentiation of both osteoblasts and osteoclasts. It has consequently become a forefront research hotspot in the field of bone metabolism [4–9].

Against this research backdrop, the number of relevant publications has grown exponentially, making it difficult for traditional literature review methods to objectively and quantitatively delineate the knowledge structure and evolutionary trajectory of the field. Bibliometrics has emerged in response to this need. As a quantitative analysis method based on large‐scale literature data, it can accurately identify research trends, collaborative networks, and emerging frontiers within a discipline [10]. Among various tools, bibliometric analysis tools such as CiteSpace, a Java‐based application for visualising knowledge domains, plays a significant role in scientific literature analysis. It integrates multiple methods, including cluster analysis, social network analysis, and multidimensional scaling analysis. By generating intuitive knowledge domain maps (mapping knowledge domain, MKD), it clearly presents the structural panorama and developmental dynamics of a scientific field. Currently, this tool is widely used to detect disciplinary frontiers and research evolution paths and has been effectively applied in numerous fields, including medicine, education, and information science [11].

Based on the aforementioned issues, we employed such methods to conduct a preliminary analysis of the OP–ATG field over the past two decades. We detected a strong emerging signal: lipid peroxidation (LP). LP is a chain reaction initiated when reactive oxygen species (ROS) attack polyunsaturated fatty acids in cell membranes. The reactive aldehydes produced (such as MDA and 4‐HNE) are not only cytotoxic but can also act as signalling molecules that profoundly regulate cell fate [12–15]. However, in current bone metabolism research, LP is predominantly situated within the research framework of ‘ferroptosis’, where it is regarded as a hallmark event of this iron‐dependent form of cell death [14, 16]. While this research paradigm has undoubtedly deepened our understanding of ferroptosis, it has inadvertently obscured LP’s potential role as a fundamental and independent biological process (BP), potentially possessing its own unique regulatory network in OP that operates independently of ferroptosis.

**Figure 1:**
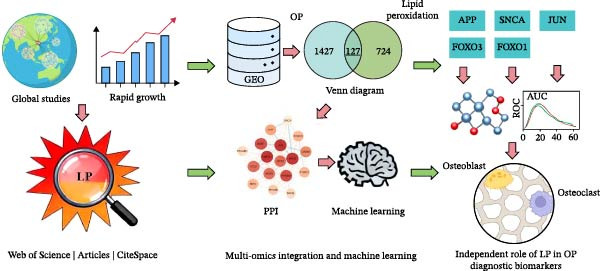
From macroscopic bibliometric analysis to microscopic gene identification.

To address this knowledge gap, this study innovatively adopts an integrated strategy that combines bibliometrics and bioinformatics. First, we employed bibliometrics to gain a macroscopic understanding of field trends and validate the scientific hypothesis that ‘LP’ represents an independent frontier. Subsequently, we designed a targeted bioinformatics analysis plan. Instead of starting from ferroptosis‐related gene sets, we directly constructed a molecular bridge between OP and LP, aiming to identify their common interaction targets and core regulatory pathways systematically and ultimately reveal the independent role network of LP in OP (Figure 1).

## 2. Data and Methods

### 2.1. Bibliometric Analysis

#### 2.1.1. Data Collection and Search Strategy

**Figure 2:**
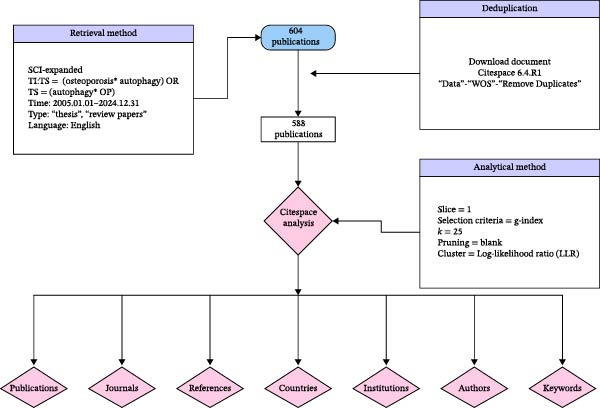
Flowchart of literature search, screening, and analysis.

The literature data for this clinical study were sourced from the Science Citation Index Expanded (SCI‐EXPANDED) within the Web of Science Core Collection database (http://apps.webofknowledge.com). The search query was TS = (osteoporosis  ^∗^ autophagy) OR TS = (autophagy  ^∗^ OP). The publication date range was set from January 1, 2005, to December 31, 2024. The document types selected were “Article” and “Review Article”, and the language was restricted to English. Articles meeting the following criteria were excluded: (1) those not formally published; (2) conference abstracts, proceedings, or errata; (3) duplicate publications. The search for this study was conducted on 26th April 2025. The initial search yielded 604 publications. After screening based on the above criteria, 588 articles were selected for further review. All selected records were exported in plain text format, with filenames following the ‘download_ ^∗∗^.txt’ pattern. These files were then imported into the CiteSpace.6.4.R1 software. The ‘Remove Duplicates’ function, located in the control panel under ‘Data’ ‐> ‘Wos’, was used for deduplication. After this process, 588 articles remained and were used for subsequent analysis (Figure 2).

#### 2.1.2. Bibliometric Analysis and Visualisation

This study utilised the software tool CiteSpace.6.4.R1 for data visualisation processing. When analysing the author collaboration networks, institutional collaboration networks, country collaboration networks, cited journals, keywords, and clusters of OP and ATG‐related literature, the relevant parameters were set as follows: Time Slicing (Slice = 1), Selection Criteria chosen as the g‐index with *k* = 25, and Pruning set to none. For the cluster analysis, the log‐likelihood ratio (LLR) algorithm was selected. The LLR algorithm is a specific algorithm within CiteSpace, capable of detailing the nature of clusters while providing optimal results regarding the uniqueness and coverage of cluster‐related topics [17].

### 2.2. Bioinformatics Analysis

#### 2.2.1. Screening and Identification of Targets Related to OP and the Key Burst Term LP

Using ‘Osteoporosis’ as the keyword, a search was conducted in the NCBI Gene Expression Omnibus (GEO) database (https://www.ncbi.nlm.nih.gov/geo/). Datasets GSE230665, GSE35956, and GSE56815 were designated as the training set, while GSE2208, GSE7429, and GSE7158 served as the validation sets. The ComBat method from the Sva package in R 4.4.2 was used to correct for batch effects between the GSE230665, GSE35956, and GSE56815 datasets [18]. The limma and ggplot2 packages were utilised to analyse differentially expressed genes (DEGs) between postmenopausal osteoporosis (PMO) and healthy controls in the training set, using a threshold of |logFC| > 0.2 and *p* < 0.05 [19]; a volcano plot of the DEGs was also generated. The keyword ‘LP’ was searched in the Genecards database to predict its associated targets. A relevance score ≥5 was set as the threshold to filter the LP target set.

#### 2.2.2. Protein–Protein Interaction (PPI) Construction and GO/KEGG Enrichment Analysis

The OP–LP intersection targets were imported into the STRING database (https://cn.string-db.org/) [20], with the species limited to *‘Homo sapiens*’ and a confidence score threshold set to ≥0.400. The results were saved in TSV format and imported into Cytoscape 3.10.1 software to construct a PPI network. The MCODE plugin was used to identify core gene clusters within the intersection targets, applying the following criteria: Degree Cutoff = 2, Node Score Cutoff = 0.2, K‐core = 2, and Max. Depth = 100. Subsequently, these targets were submitted to the DAVID (https://davidbioinformatics.nih.gov/) online platform for GO and KEGG enrichment analysis, specifying *Homo sapiens* as the target species [21]. The GO enrichment analysis primarily included BPs, cellular components (CC), and molecular functions (MF). The top 5 most significant GO terms and the 15 KEGG pathways most strongly associated with OP and LP were selected. These results were visualised using the Microbioinfo online platform, presented as bubble charts and bar graphs.

#### 2.2.3. Construction and Analysis of Machine Learning Models

To further identify feature genes associated with OP–LP, this study constructed 113 models based on combinations of 12 machine learning methods, including LASSO, Ridge, Enet, StepGlm, SVM, glmboost, LDA, plsRglm, Random Forest, GBM, XGBoost, and Naive Bayes, to better predict candidate targets. The predictive performance of the optimal models for both the training set and validation sets is presented using confusion matrices.

#### 2.2.4. Screening and Validation of Key Targets

Candidate targets derived from the best‐performing machine learning model were intersected with the nodes of the MCODE core gene cluster. The resulting overlapping targets were considered as key targets through which LP influences OP. Subsequently, we analysed the interrelations between these genes in the training set and the validation set, respectively. Receiver operating characteristic (ROC) curves were constructed, and the expression levels of key targets in the OP group were compared to those in the control group. This analysis was used to evaluate the diagnostic specificity and sensitivity of the key targets and to appraise their predictive efficacy.

## 3. Results

### 3.1. General Characteristics of the Literature

#### 3.1.1. Annual Publication Volume and Citation Frequency

**Figure 3:**
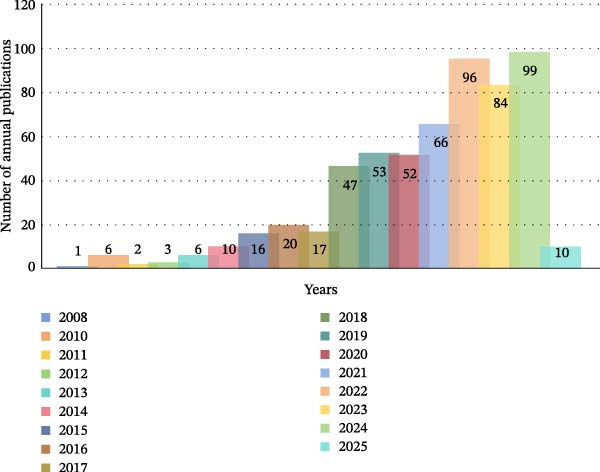
Annual publication trend of OP–ATG‐related research literature over the past 20 years.

As some articles from 2024 were published in 2025, they were still categorised within the 2024 timeframe for this analysis. Within the search period, the earliest published article related to OP–ATG was ‘Sodium Nitroprusside Induces Autophagic Cell Death in Glutathione‐Depleted Osteoblasts’ by Son, MJ, Lee, SB, et al., published on 1st January 2010 in the ‘Journal of Biochemical and Molecular Toxicology’. Figure 3 reflects the research activity and development trends in the field of OP and ATG over the past two decades. As shown in the figure, no relevant research papers were published between 2005 and 2007. From 2008 to 2017, the annual publication volume in OP and ATG‐related fields showed a year‐on‐year increasing trend, albeit with a modest increment. This indicates that research on OP and ATG was in its initial stages during this period. Driven by global socioeconomic development, rising public living standards, and increasing healthcare demands, the annual publication volume in this field saw a substantial rise in 2018 (47 articles). Subsequently, it maintained a notably high and increasing trend, with an average of 72.43 articles per year (including 2025 publications within the 2024 count). This demonstrates the growing emphasis placed on OP and ATG research by both clinical and academic communities over the last 20 years. Thereafter, the field entered a period of high‐volume growth, a phenomenon potentially linked to mature global collaboration and research models, as well as the escalating challenge of global ageing. We speculate that future developments in the field may progress towards personalised therapies targeting ATG and AI‐driven drug design.

The top five most cited publications from the 588 articles are presented in Table 1. The most cited work was ‘Autophagy—a key player in cellular and body metabolism’ by Kim et al. (2014), with citation counts reaching 750. This publication primarily emphasised the influence of nutrients on the regulation of ATG, and further described the role of ATG in regulating energy metabolism and in the development of diseases associated with metabolic alterations.

**Table 1:** Top five most cited publications from the 588 Articles.

Rank	Count	Cited references	Authors/Year
1	750	Autophagy—a key player in cellular and body metabolism	Kim, KH/2014
2	608	mTOR signalling pathway and mTOR inhibitors in cancer: progress and challenges	Zou, ZL/2020
3	377	Identification of senescent cells in the bone microenvironment	Farr, JN/2016
4	322	Autophagy in osteoblasts is involved in mineralisation and bone homeostasis	Nollet, M/2014
5	321	What old means to bone	Manolagas, SC/2010

#### 3.1.2. Co‐Cited Journals

The co‐cited journals were subjected to cluster visualisation using the LLR algorithm in the bibliometric analysis (Figure 4A). The co‐cited journals were divided into 7 clusters (numbered 0–6). In the resulting map, different coloured areas represent different clusters. The size of a cluster module is inversely proportional to its cluster number; that is, a smaller module has a larger number, indicating that cluster #0 represents the largest cluster. The cluster modules derived from the analysis serve as a global measure of the overall structure. The modularity (*Q*) value and the Weighted Mean Silhouette (*S*) value are key metrics for evaluating the overall structural performance of the map. A *Q*‐value > 0.3 indicates effective clustering within the map, and an *S*‐value >0.5 signifies that the cluster analysis results are credible. As shown, the co‐cited journal network comprises *N* = 557 nodes and *E* = 5249 links, with a density of 0.0339. The *Q*‐value was 0.2791 (<0.3), and the *S*‐value was 0.6308 (>0.5). The modularity *Q*‐value, while slightly below the conventional threshold of 0.3, should be interpreted in conjunction with the high silhouette *S*‐value. A *Q*‐value > 0.3 suggests a significant community structure, whereas our observed value of 0.2791 indicates a network that is more interconnected with less rigidly defined boundaries between clusters. This pattern is highly indicative of a highly cross‐disciplinary and integrated research field [22]. The high *S*‐value confirms that the clusters we did identify are internally coherent and reliable. Therefore, the combination of a moderately low *Q* and a high *S* likely reflects the reality that journals in the OP and ATG field frequently cite each other across sub‐topics (e.g., pharmacology, molecular biology, and clinical medicine), forming a cohesive knowledge domain rather than isolated silos. This integrative nature is a hallmark of contemporary biomedical research. The largest module was #0 Pharmacological effect, followed by others such as #1 OVX mice and #2 Rankl‐induced osteoclast precursor A. This indicates that, over the past two decades, the journals most frequently co‐cited by articles related to OP and ATG have primarily focused on areas such as pharmacological effects and OVX mice. The top five most cited co‐cited journals are shown in Figure 4B. In descending order of co‐citation frequency, these journals were: ‘Journal of Bone and Mineral Research’, ‘Autophagy’, ‘Journal of Biological Chemistry’, ‘Bone’, and ‘Nature‘. Thus, these journals collectively form the core knowledge framework of the OP and ATG field. They provide the theoretical foundation and a translational bridge for elucidating the pathological mechanisms of ATG‐oxidative stress in regulating bone homeostasis, advancing targeted drug therapies (e.g., pharmacological studies based on OVX mouse models), and developing regenerative medicine strategies, thereby establishing a comprehensive research chain from molecular mechanisms to clinical intervention.

Figure 4Overview of publishing journals and co‐cited journals in the OP and ATG research field. (A) Cluster analysis diagram of co‐cited journals. (B) Top 5 most cited co‐cited journals. (C) Overlay path diagram of publishing journals and co‐cited journals.

**Figure:**
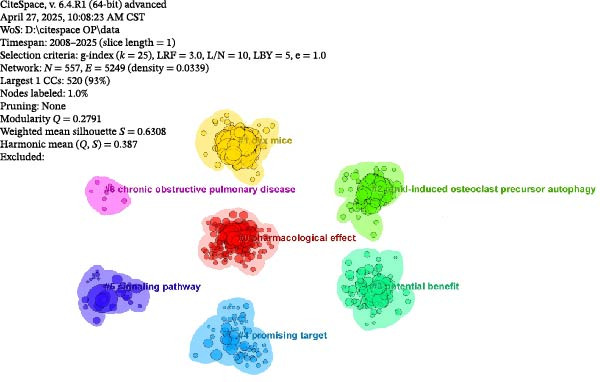
(A)

**Figure:**
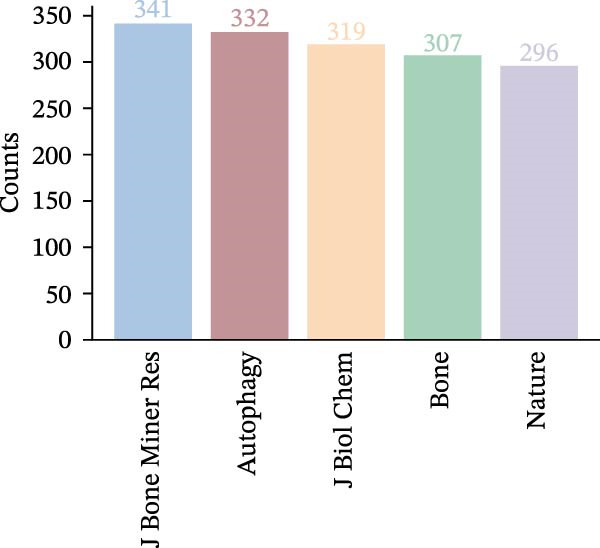
(B)

**Figure:**
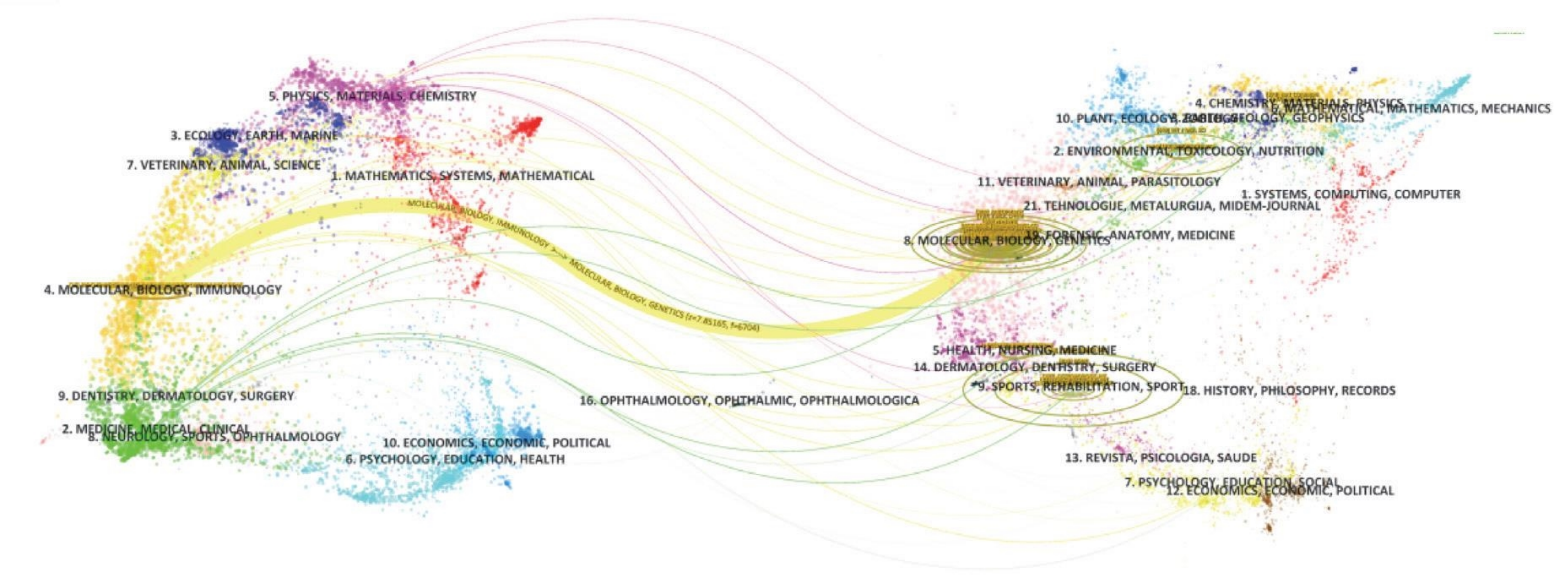
(C)

Furthermore, we employed the dual‐map overlay function of CiteSpace to construct the thematic distribution of academic journals (Figure 4C). For each published article, the journal in which it is published resides in the citing journal network on the left side of the base map. The references cited in the article are located in the cited journal network on the right side of the base map. The trajectory of a collection over time is displayed retrospectively as a path formed by the weighted centres of the corresponding citing publications and their cited references. The dynamics of fundamental research activities can be revealed through the stability of the corresponding trajectories [23]. As shown in the figure, the dual‐map overlay of journals primarily reveals two main citation paths. The journals publishing the citing articles are predominantly concentrated in fields such as ‘MATHEMATICS‘, ‘SYSTEMS‘, ‘MATHEMATICAL‘, ‘MEDICINE‘, ‘MEDICAL‘, and ‘CLINICAL‘. Conversely, the cited references were primarily published in journals within fields such as ‘SYSTEMS‘, ‘COMPUTING‘, ‘COMPUTER‘, ‘ENVIRONMENTAL‘, ‘TOXICOLOGY‘, and ‘NUTRITION‘.

#### 3.1.3. Co‐Cited References

**Figure 5:**
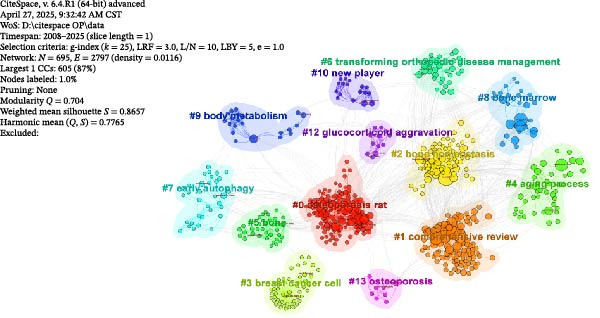
Cluster analysis of co‐cited references in the OP and ATG research field.

Subsequently, we analysed and performed cluster network analysis on the co‐cited references from articles published in the OP and ATG field over the past two decades (Figure 5). According to the figure, the network of co‐cited references consists of *N* = 695 nodes and *E* = 2797 links, with a density of 0.0116. The *Q*‐value was 0.704 (>0.3), and the *S*‐value was 0.8657 (>0.5), indicating a significant and credible cluster structure. The co‐cited references from the last 20 years could be divided into 14 clusters (numbered 0–13), with the exception of cluster #11. The co‐cited references were primarily concentrated in clusters such as #0 Osteoporosis in rats, #1 Comprehensive review, and #2 Bone homeostasis.

The co‐cited references in the OP and ATG field were ranked in descending order based on citation frequency and centrality, with the top 5 entries presented in Tables 2 and 3, respectively. Node Centrality was calculated to identify nodes situated between two or more major node groups. In a network, a node with a Centrality greater than 0.1 (i.e., a node interconnected with more than 10% of the other nodes) exerts a significant influence on other nodes, as a greater volume of information is channelled through it [24]. The top five most co‐cited articles delve into the immense potential of ‘ATG‘ as a core regulator of skeletal health and a novel therapeutic target for OP, traversing from basic biology (ATG) to cellular functions (stem cells, osteoblasts), then to pathological mechanisms (ageing, stress), and finally to disease application (OP treatment). Furthermore, the top five articles ranked by co‐citation centrality all focus on the clinical medication for PMO patients. These articles indicate that Denosumab, Teriparatide, and Romosozumab are commonly used drugs in the clinical treatment of OP, demonstrating relatively ideal clinical therapeutic efficacy. Thus, these publications also provide a scientific basis and support for the research and development in this field.

**Table 2:** Top five most co‐cited references in OP and ATG research.

Rank	Count	Cited references	Authors/Year
1	56	Autophagy in bone homeostasis and the onset of osteoporosis	Yin X/2019
2	39	Targeting autophagy in osteoporosis: from pathophysiology to potential therapy	Li X/2020
3	37	Autophagy controls mesenchymal stem cell properties and senescence during bone ageing	Ma Y/2018
4	35	Defective autophagy in osteoblasts induces endoplasmic reticulum stress and causes remarkable bone loss	Li HX/2018
5	34	The role of autophagy and mitophagy in bone metabolic disorders	Wang S/2020

**Table 3:** Top five co‐cited references by centrality in OP and ATG research.

Rank	Centrality	Cited References	Authors/Year
1	0.1	2 years of Denosumab and teriparatide administration in postmenopausal osteoporosis women with osteoporosis (The DATA Extension Study): a randomised controlled trial	Leder BZ/2014
2	0.07	Romosozumab in postmenopausal osteoporosis women with low bone mineral density	Mcclung MR/2014
3	0.07	Denosumab and teriparatide transitions in postmenopausal osteoporosis (the DATA‐Switch study): extension of a randomised controlled trial	Leder BZ/2015
4	0.06	Relationship between bone mineral density changes with denosumab treatment and risk reduction for vertebral and nonvertebral fractures	Austin M/2012
5	0.05	10 years of denosumab treatment in postmenopausal osteoporosis women with osteoporosis: results from the phase 3 randomised FREEDOM trial and open‐label extension	Bone HG/2017

### 3.2. Bibliometric Analysis of Collaboration Patterns

#### 3.2.1. National Collaboration Visualisation

A collaboration network analysis was conducted for countries involved in OP and ATG‐related research (Figure 6A), with the top 10 countries by publication volume highlighted on a world map (Figure 6B). In the figures, each node represents a country, where the node radius is positively correlated with publication count, and connecting lines indicate some level of collaboration or connection between countries. This convention applies to all subsequent similar figures. As shown in Figure 6A, the national network comprises *N* = 44 nodes and *E* = 80 links, with a density of 0.0846. Collaboration between China and the USA, China and South Korea, and China and Germany was relatively close. However, domestic collaborations still predominated overall, indicating there remains room for enhancement in transnational collaboration. Figure 6C, D shows that China had the highest number of publications, while the USA had the highest centrality. China’s publication count (434) was significantly higher than that of the second‐ranked USA (72); the Centrality of the USA (0.66) was slightly higher than that of China (0.63). This landscape reflects China’s dominant role in the quantitative output within this field, while institutions in Europe and America still maintain advantages in theoretical innovation and technological leadership.

Figure 6National contribution landscape in OP and ATG research. (A) National collaboration network. (B) Geographical distribution of top 20 countries by publication volume. (C) Top 5 countries by publication count. (D) Top 5 countries by centrality.

**Figure:**
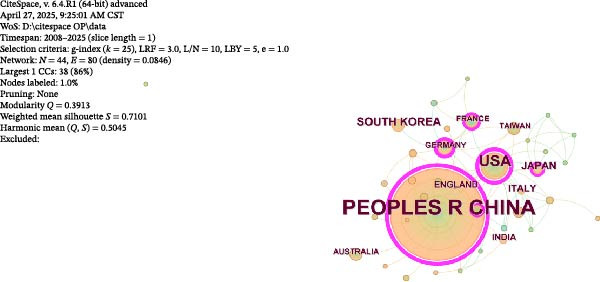
(A)

**Figure:**
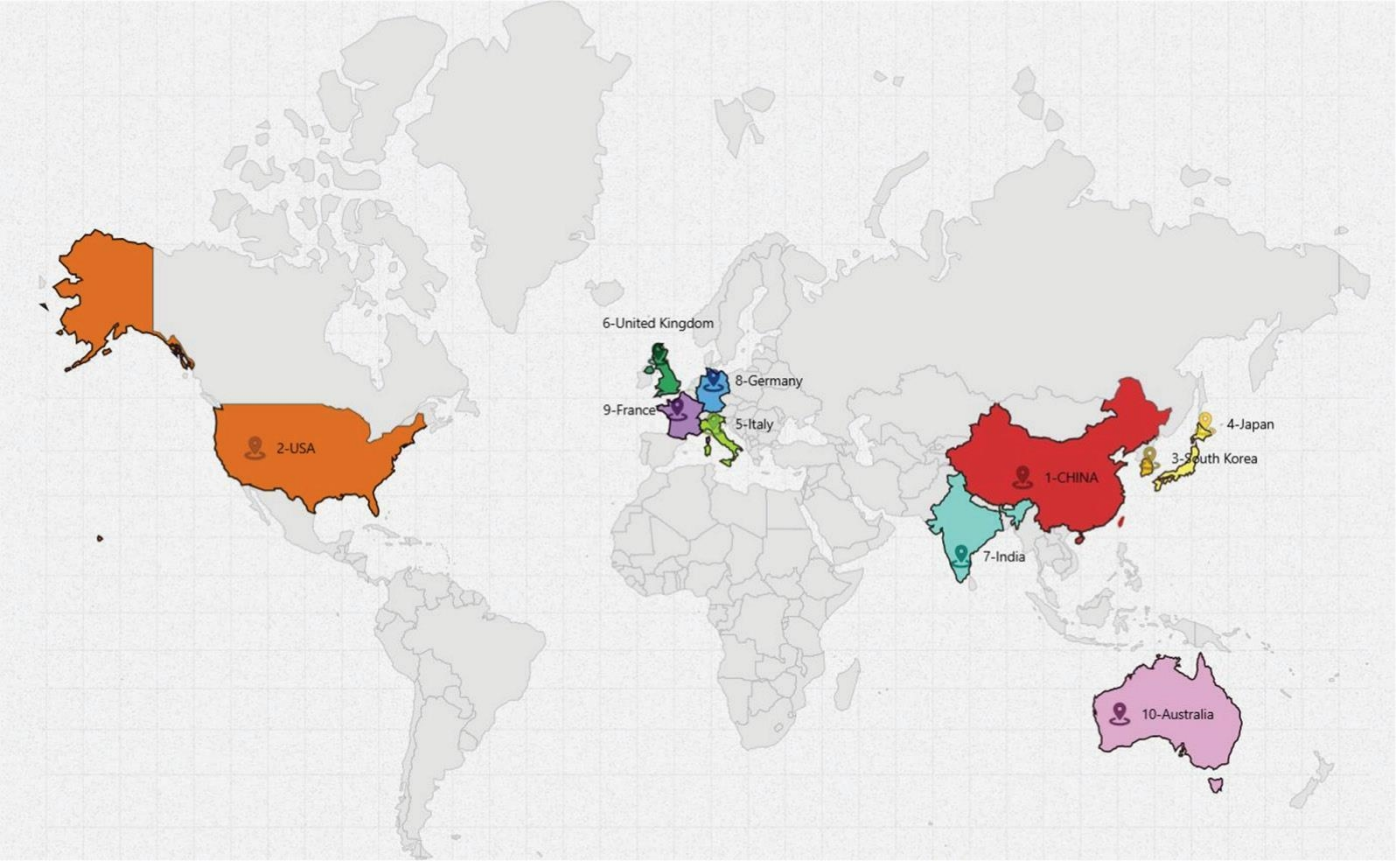
(B)

**Figure:**
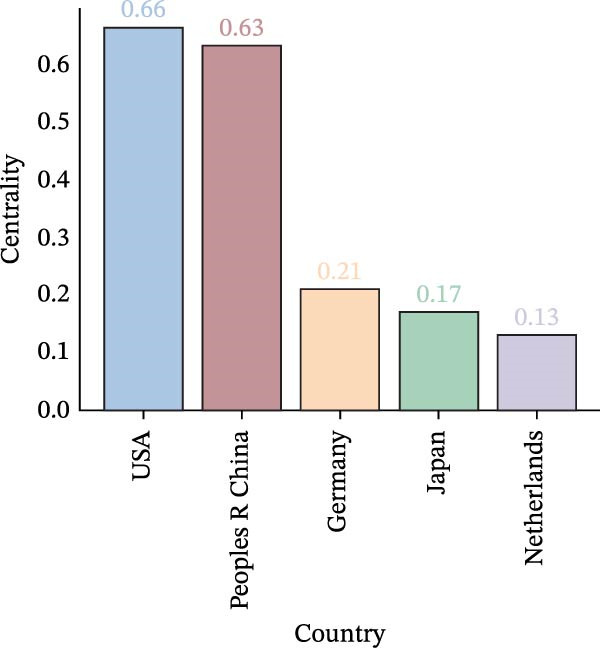
(C)

**Figure:**
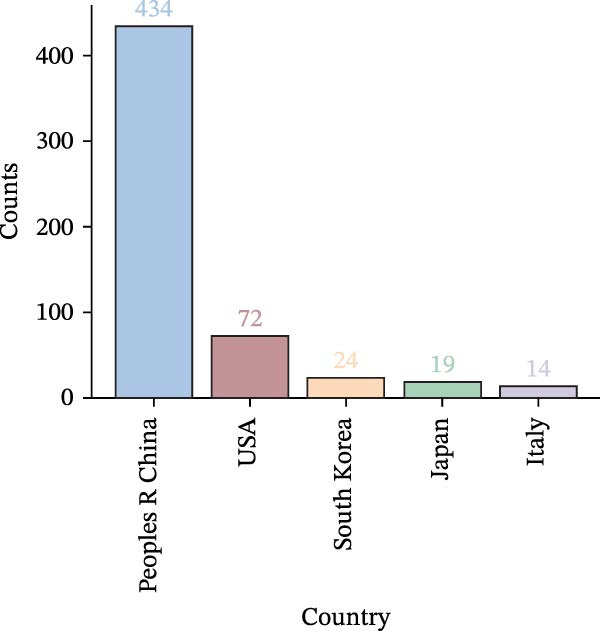
(D)

#### 3.2.2. Institutional Collaboration Visualisation

A visual analysis of collaborative relationships among institutions engaged in OP and ATG‐related research was conducted (Figure 7A). As shown, the institutional network comprises *N* = 301 nodes and *E* = 351 links, with a density of 0.0078. The number and density of links represent the connections between institutional nodes; a greater number and higher density of links indicate closer inter‐institutional relationships. The figure demonstrates that research institutions have maintained relatively close and well‐connected collaborations in the OP and ATG field over the past two decades. The top five institutions by publication count and the top five by centrality are presented in Figure 7B, C. The institution with the highest number of publications was China Medical University (24 articles). The institution with the highest centrality was the Chinese Academy of Sciences (0.08). All institutions in the top five for both publication volume and centrality were from China. This indicates China’s dominant position in research within this field, likely attributable to substantial national investment in biomedical research and concentrated efforts by researchers in the area. Furthermore, a domestic collaboration network has formed, centred around leading institutions (such as China Medical University and the Chinese Academy of Sciences), facilitating knowledge sharing and technology transfer. However, the current landscape highlights a shortcoming in international participation. Future efforts should focus on strengthening transnational collaboration to transform regional advantages into global influence and enhance the universality of research outcomes.

Figure 7Institutional landscape in OP and ATG research. (A) Institutional collaboration network. (B) Top 5 institutions by publication count. (C) Top 5 institutions by centrality.

**Figure:**
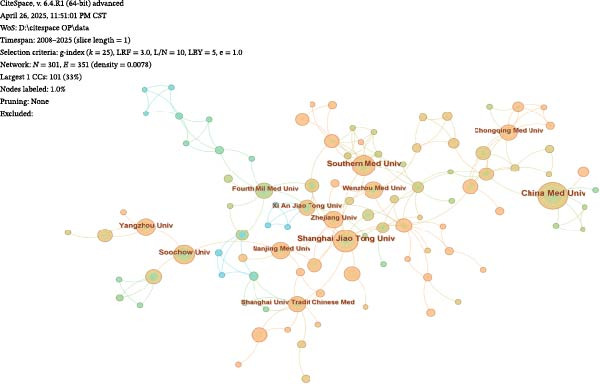
(A)

**Figure:**
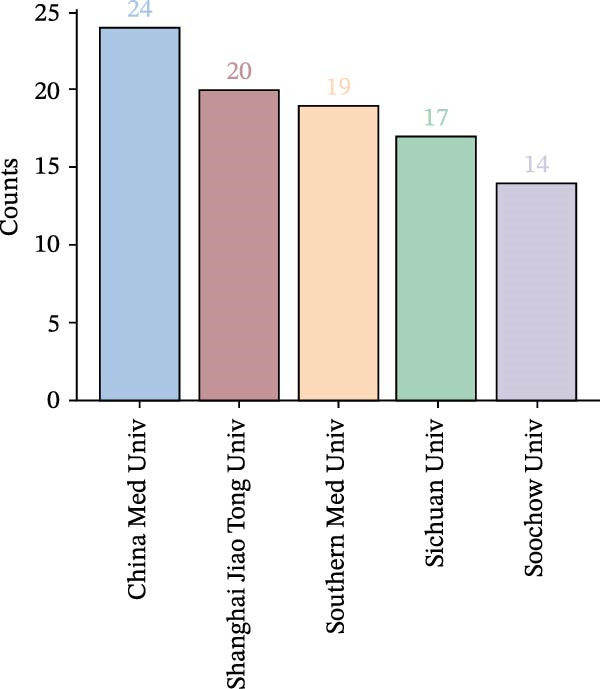
(B)

**Figure:**
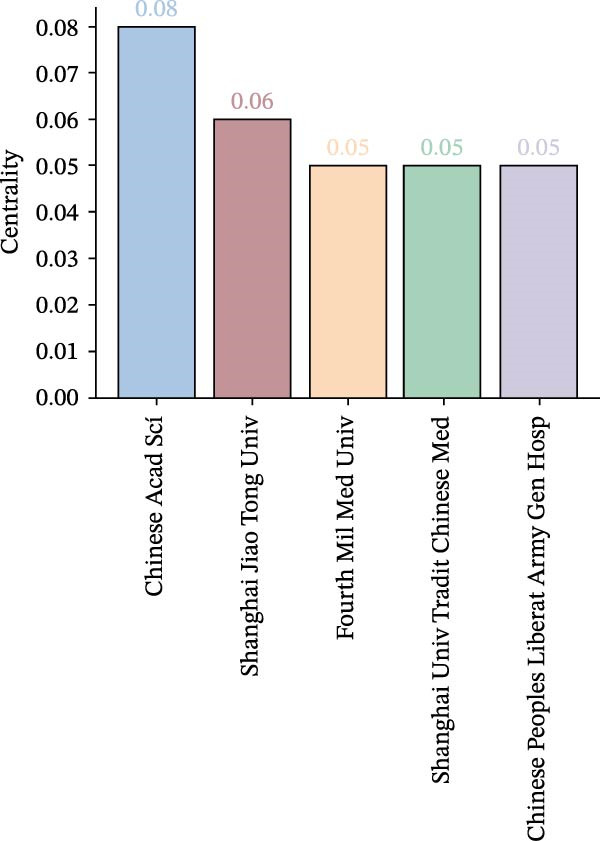
(C)

#### 3.2.3. Author and Co‐Cited Author Collaboration Visualisation

A collaboration network analysis was conducted for authors publishing in the OP and ATG field (Figure 8A). In the figure, each node represents an author, where the node radius is positively correlated with publication count. Connecting lines between nodes indicate some degree of collaboration or connection between authors, with line thickness positively correlated with the strength of the relationship. As shown, the author network comprises *N* = 467 nodes and *E* = 920 links, with a density of 0.0085. The analysis reveals that the OP and ATG field has formed multiple collaborative teams, represented by researchers such as Liu, Zongping, Song, Ruilong, and Bian, Jianchun and among others. However, most teams lacked collaborative connections between them, with cooperation largely confined within individual teams.

Figure 8Author and co‐cited author landscape in OP and ATG research. (A) Author collaboration network. (B) Top 5 authors by publication count. (C) Co‐cited author collaboration network. (D) Top 5 most co‐cited authors.

**Figure:**
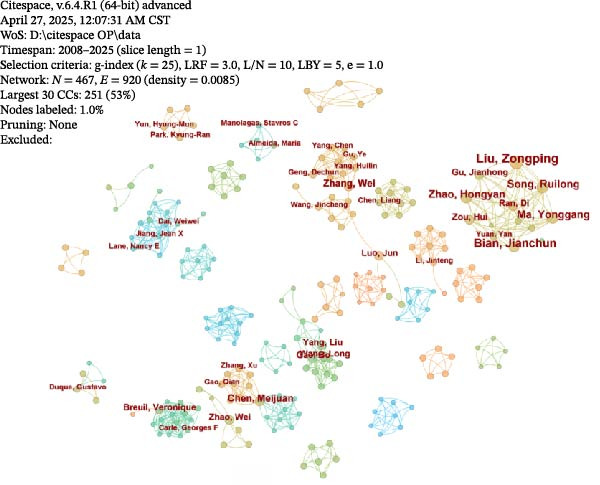
(A)

**Figure:**
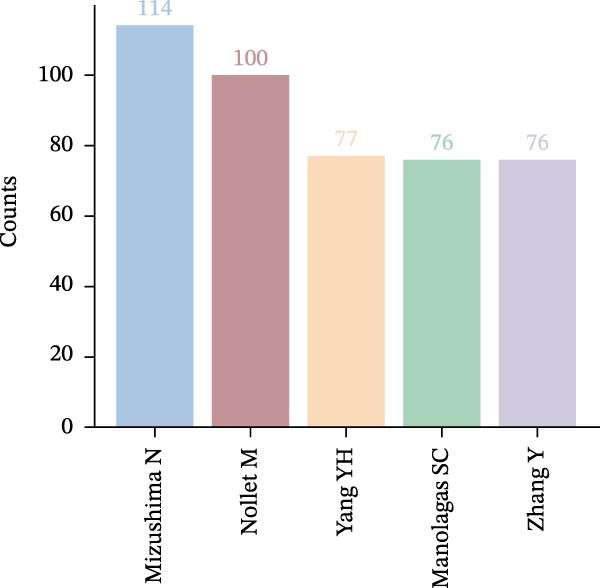
(B)

**Figure:**
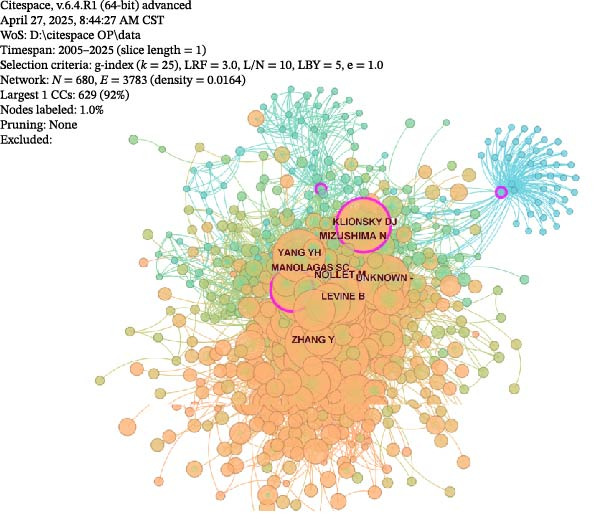
(C)

**Figure:**
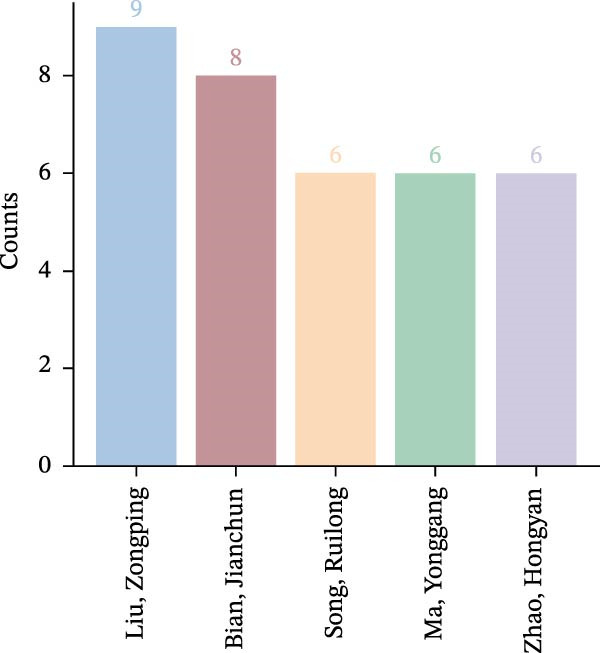
(D)

Figure 8B shows the top five authors by publication count in the OP and ATG field. The most prolific author was Liu, Zongping, who published nine articles over the past two decades, with the first publication in 2016. This was followed by Bian, Jianchun, with eight publications.

A visual network analysis was performed on co‐cited authors in the OP and ATG field (Figure 8C). As shown, the co‐cited author network comprises *N* = 680 nodes and *E* = 3783 links, with a density of 0.0164. Figure 8D displays the top five most co‐cited authors in the OP and ATG field. The most frequently co‐cited author was Professor Noboru Mizushima from The University of Tokyo, co‐cited 114 times within the study period. Professor Noboru Mizushima is an internationally recognised authority in ATG research, focusing primarily on the molecular mechanisms of ATS and its role in cellular homeostasis. He has made pioneering contributions, particularly in understanding autophagosome formation and elucidating the functions of ATG‐related gene families [25, 26]. Although Professor Mizushima’s work does not directly investigate OP, his foundational research has provided crucial molecular tools and mechanistic frameworks for the OP field. For instance, Atg5/Atg7 conditional knockout mice, developed from his research, are widely used to study ATG function in bone cells, revealing that ATG deficiency leads to impaired osteoblast function or excessive osteoclast activation, subsequently causing bone loss. Thus, his work has indirectly advanced the pathological understanding of ATG in bone metabolic imbalances (such as OP and osteoarthritis) and the exploration of therapeutic strategies. His contributions have established the theoretical foundation for the research direction of ‘targeting ATG to regulate bone remodelling‘.

### 3.3. Keyword Visual Analysis

#### 3.3.1. Keyword Co‐Occurrence Analysis

Co‐occurrence analysis of keywords from OP and ATG‐related literature can reveal the research focus within this field, thereby providing a reliable basis and support for subsequent clinical research and development. Following the method described in Section 2.2, a co‐occurrence analysis was performed on the keywords from the 588 articles in this field (Figure 9A). As shown, the keyword network consists of *N* = 641 nodes and *E* = 2622 links, with a density of 0.0247. In the figure, the node size is positively correlated with the frequency of the keyword. Nodes with high betweenness centrality are encircled in purple, while nodes with high burst strength feature a red inner ring. The top 10 keywords by frequency and the top 10 by centrality were selected for statistical analysis (Supporting Information 1: Table S1).

Figure 9Keyword landscape in the OP and ATG research field. (A) Keyword co‐occurrence network. (B) Keyword cluster analysis.

**Figure:**
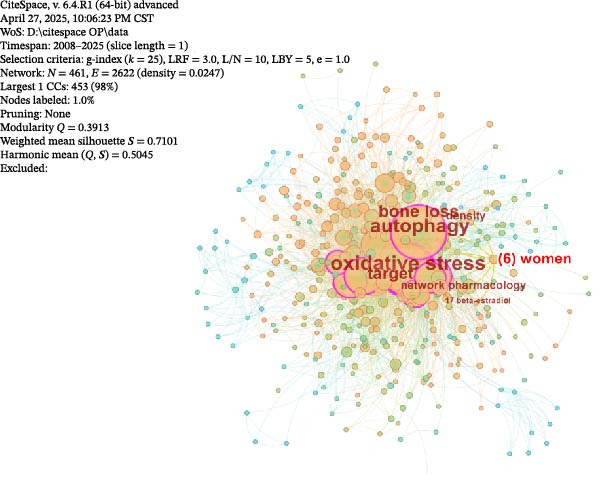
(A)

**Figure:**
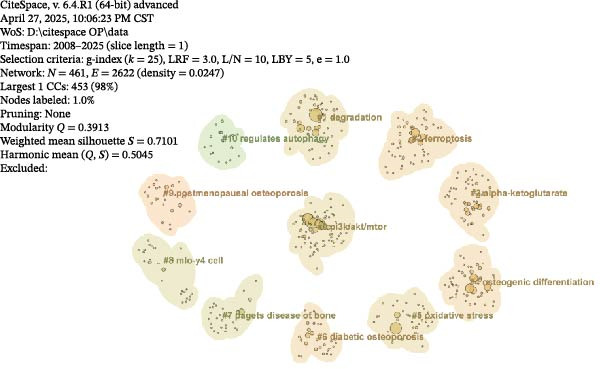
(B)

As shown in Supporting Information 1: Table S1, ‘ATG’ was the most frequent keyword, occurring 180 times, followed by ‘Oxidative stress’ (144), ‘OP’ (115), ‘Differentiation’ (93), ‘Apoptosis’ (87), and others. ‘Oxidative stress’ (0.27) had the highest centrality, followed by ‘ATG’ (0.18), ‘NF kappa B’ (0.16), ‘Mesenchymal stem cells’ (0.13), and ‘Expression’ (0.13), amongst others. Analysis of the highest‐frequency keywords reveals that they predominantly involve fields such as cell and molecular biology, bone metabolism, and stem cells and regenerative medicine. Research data from the past two decades demonstrate that ‘ATG’ and ‘Oxidative Stress’ occupy a dominant position as core keywords in the OP field. Together, they reveal the pathological core of bone metabolic imbalance: ATG, as a key mechanism regulating cellular homeostasis, maintains the osteoblast/osteoclast balance by clearing damaged organelles (e.g., mitochondria); oxidative stress, through the accumulation of ROS, leads to osteocyte apoptosis and impaired differentiation of mesenchymal stem cells, acting as an ‘accelerator’ of OP progression [6, 7, 27, 28].

#### 3.3.2. Keyword Cluster Analysis

Cluster analysis of the keywords from the included research literature can, to a certain extent, reflect research trends regarding ATG in the field of OP. The number of keywords within a cluster module (reflecting the module’s scope and size) indicates its relative importance, which is beneficial for elucidating the field’s developmental patterns and directions. Cluster analysis of the keywords was performed, ultimately yielding 11 relevant clusters (numbered 0–10), which are presented in the module analysis diagram (Figure 9B). As shown, the *Q*‐value was 0.3913 (>0.3) and the *S*‐value was 0.7101 (>0.5), indicating that the reliability of this cluster analysis is high. From the figure, it is evident that clusters such as #0 PI3K/AKT/MTOR, #1 Degradation, and #10 Regulates ATG primarily pertain to ATG regulatory mechanisms. This dimension focuses on the molecular pathways and regulatory processes of ATG. The PI3K/AKT/MTOR signalling pathway is a core regulator of ATG. At the same time, terms like ’Regulates autophagy’ and ’Degradation’ directly relate to the initiation and execution phases of ATG, influencing cellular homeostasis and bone metabolism in OP.

Furthermore, clusters #2 Ferroptosis, #3 Ketoglutarate, and #5 Oxidative stress encompass cellular stress and metabolism related to ATG. Ferroptosis is an iron‐dependent form of cell death, oxidative stress involves the accumulation of ROS, and ketoglutarate, as a metabolite, participates in cellular energy metabolism and the regulation of ATG. These processes are closely associated with bone cell damage and repair in OP.

#### 3.3.3. Keyword Burst Analysis

**Figure 10:**
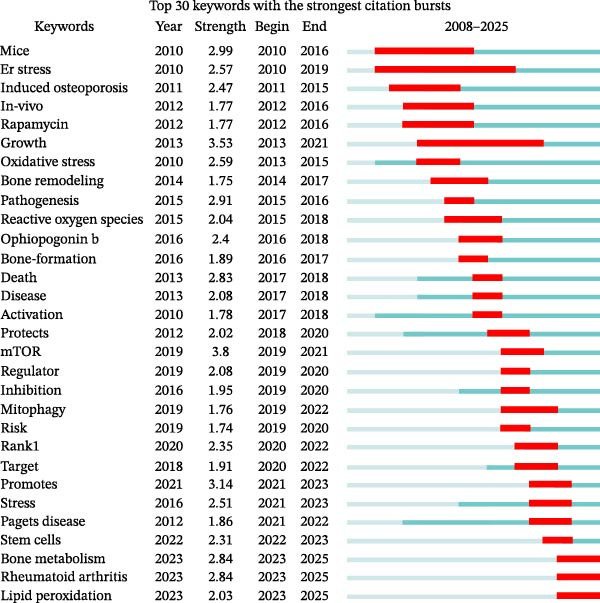
Keyword burst analysis of OP–ATG‐related literature.

Keyword bursts can, to a certain extent, reflect emerging academic trends and new topics in recent years, aiding in the prediction of frontier research directions and revealing potential hotspots within a field [29]. The ‘Burstness’ function was selected in the CiteSpace control panel, with γ set to 0.5, yielding 42 keywords. We performed a burst analysis on the top 30 of these (Figure 10). ‘Year’ indicates the year the keyword first appeared; ‘Strength’ represents the burst intensity, with higher values indicating greater influence; ‘Begin’ and ‘End’ denote the start and end years of the keyword’s burst period, respectively. This figure visually displays the historical research foci within the field, allowing for the prediction of future development trends in OP and ATG research. The figure shows that ‘ER stress’ had the longest burst duration, spanning from 2010 to 2019. Furthermore, the keywords ‘bone metabolism’, ‘rheumatoid arthritis’, and ‘LP’ emerged as burst terms in 2023 and remain research priorities.

A deeper analysis of the reasons reveals distinct phases. Between 2010 and 2016, OP and ATG research primarily focused on basic models and stress mechanisms, characterised by keywords such as ‘mice’, ‘in vivo’, ‘induced OP’, and ‘oxidative stress’, concentrating on animal model establishment and fundamental pathological mechanisms like oxidative stress and endoplasmic reticulum (ER) stress. From 2016 to 2020, research into OP and ATG regulatory mechanisms deepened, shifting focus towards molecular mechanisms and pathway analysis. Keywords like ‘mtor’, ‘mitophagy’, and ‘rankl’ became hotspots, as technological advances (e.g., gene editing) helped elucidate the crucial roles of the mTOR signalling pathway, mitophagy, and the RANKL/OPG system in bone remodelling. Therapeutics targeting mTOR (e.g., rapamycin) and RANKL inhibitors (e.g., Denosumab) gradually gained prominence. During the 2020–2024 (2025) period, multi‐omics technologies (e.g., metabolomics, single‐cell sequencing) facilitated the discovery of precise targets. Novel mechanisms, such as LP, revealed the interconnected bone‐immune‐metabolism network. Research in the OP and ATG field has transitioned from a singular disease focus to systemic regulation, gradually shifting towards emerging directions such as precise targeting and cross‐disease associations. The rise of keywords such as ‘stem cells’, ‘promotes’, ‘rheumatoid arthritis’, and ‘LP’ reflects this expansion into stem cell therapies, pro‐osteogenic strategies, and intersections with other immune‐metabolic diseases (e.g., rheumatoid arthritis).

In summary, the keyword visual analysis systematically revealed that ’LP’ is a robust emerging direction in OP and ATG research. However, the mechanism by which LP, as an independent biological response, exerts its role in the pathological process of OP by regulating specific genes and pathways remains uncharacterised, and bibliometrics alone cannot elucidate this process. To address this knowledge gap, this study employed bioinformatics and machine learning approaches to identify common interaction targets between OP and LP, and to elucidate the BPs and signalling pathways involved. This provides solid molecular biological evidence for the research frontier identified by bibliometrics, transforming the trending topic of “LP” into a concrete mechanistic theory and laying the groundwork for subsequent experimental validation and targeted therapy.

### 3.4. Bioinformatics Analysis

#### 3.4.1. Identification of OP–LP Candidate Targets

After correcting for batch effects across the GSE230665, GSE35956, and GSE56815 datasets, a total of 1554 OP‐related targets were identified using the thresholds of |logFC| >0.2 and *p* < 0.05 (Figure 11A, B). Using ’LP’ as a search term in the Genecards database and applying a Relevance score threshold of ≥5851 LP‐related targets were obtained. Subsequently, the OP and LP target lists were merged, and their intersection was identified, resulting in 127 overlapping targets (Figure 11C). These targets are considered potential factors through which LP influences the onset and progression of OP, that is, potential candidate targets; the specific targets are listed in Supporting Information 2: Table S2.

Figure 11Identification of potential targets and subsequent GO/KEGG and target organ enrichment analyses. (A) PCA plots before and after batch effect removal. (B) Volcano plot. (C) Venn diagram for identification of potential targets; Dual bar chart of the top 10 BP, MF, and CC terms by *p*‐value.

**Figure:**
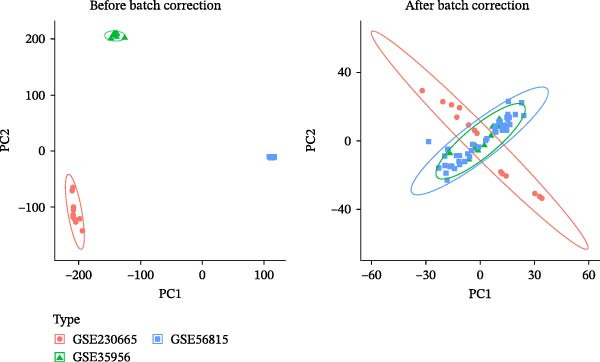
(A)

**Figure:**
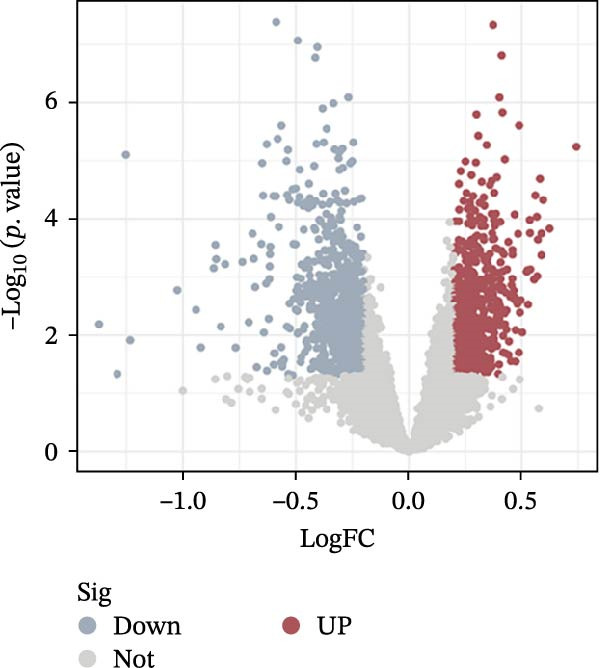
(B)

**Figure:**
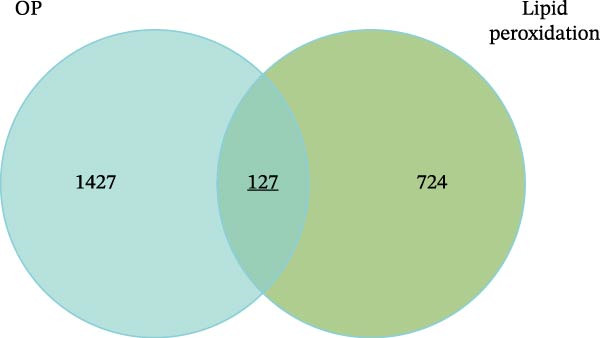
(C)

#### 3.4.2. PPI Network Construction and GO/KEGG Enrichment Analysis

The 127 potential targets were submitted to the STRING online platform, and a PPI network was constructed using Cytoscape 3.10.1 software. The network comprised 125 nodes and 911 edges, wherein the node size and colour depth are proportional to the degree value (Figure 12A). The MCODE plugin was used to identify a core gene cluster containing 17 nodes (Figure 12B). GO and KEGG enrichment analyses for the 127 potential targets were performed using the DAVID database, with *’Homo sapiens*’ set as the target species. The GO enrichment results included the three standard categories: BP, MF, and CC. In total, the 127 targets were enriched in 367 BP terms, 98 MF terms, 75 CC terms, and 96 pathways (Supporting Information 3: Table S3). The top five BP, MF, and CC terms, ranked by *p*‐value, are displayed in a bubble chart (Figure 12C). The results show that BP terms were closely associated with ‘cholesterol metabolic process’ and ‘positive regulation of interleukin‐6 production’, amongst others. CC terms were significantly related to the ‘extracellular region’, ‘extracellular exosome’, and ‘extracellular space’. MF terms were predominantly linked to ‘enzyme binding’, ‘phospholipid binding’, and ‘lipid binding’. Furthermore, a bar chart (Figure 12D) displays 15 pathways significantly associated with OP/LP, including the AGE‐RAGE signalling pathway in diabetic complications, the NF‐κB signalling pathway, the TNF signalling pathway, and neutrophil extracellular traps (NETs), along with their corresponding enriched genes.

Figure 12PPI network of potential targets and GO/KEGG enrichment results. (A) PPI network of the 127 potential targets, where node colour depth and size are proportional to the Degree value; (B) Node network diagram of the MCODE core gene cluster; (C) Bubble chart of the top 5 BP, MF, and CC terms by P‐value; (D) Bar plot showing the top 15 enriched KEGG pathways ranked by *p*‐value, along with their associated genes.

**Figure:**
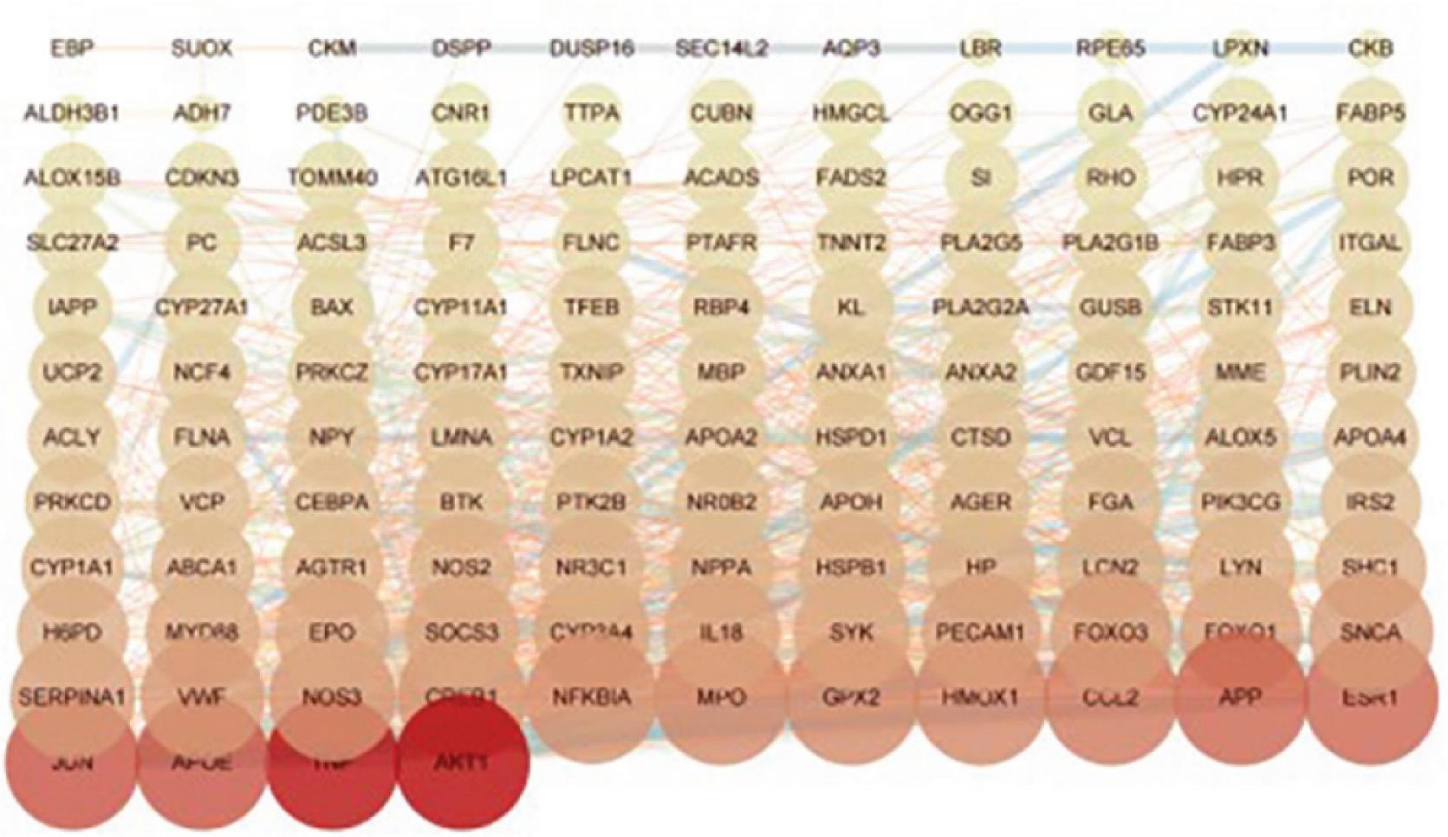
(A)

**Figure:**
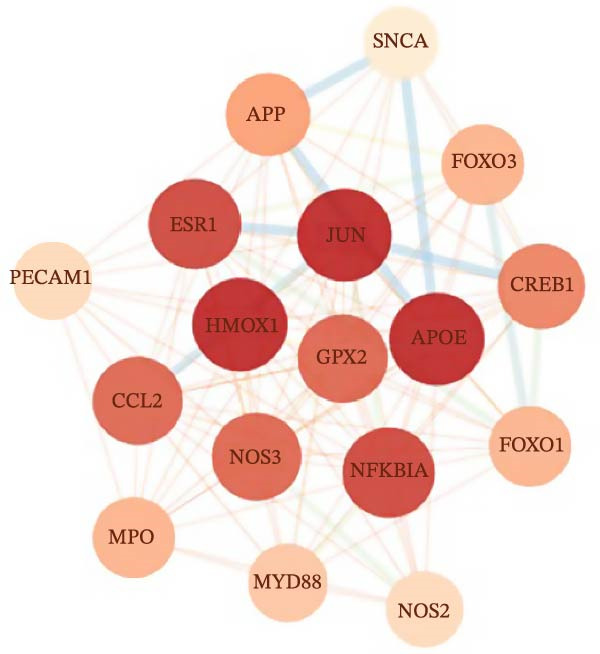
(B)

**Figure:**
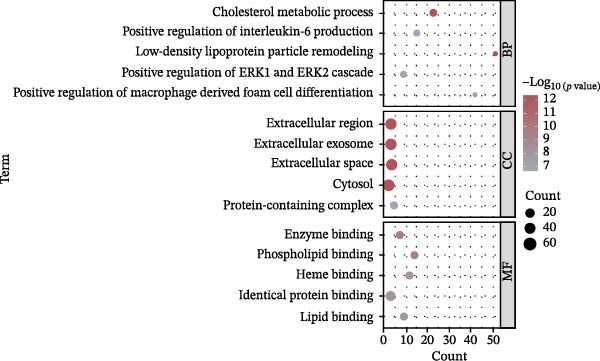
(C)

**Figure:**
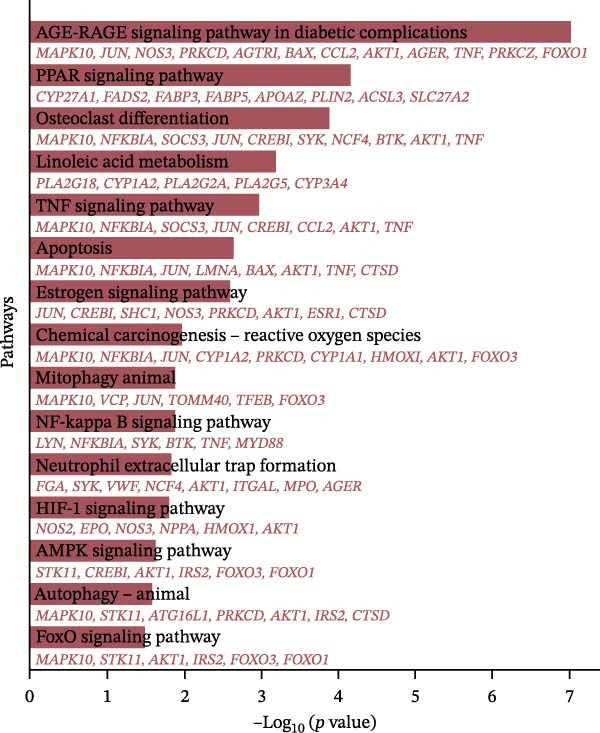
(D)

#### 3.4.3. Identification of Candidate Targets Based on Multiple Machine Learning Models

**Figure 13:**
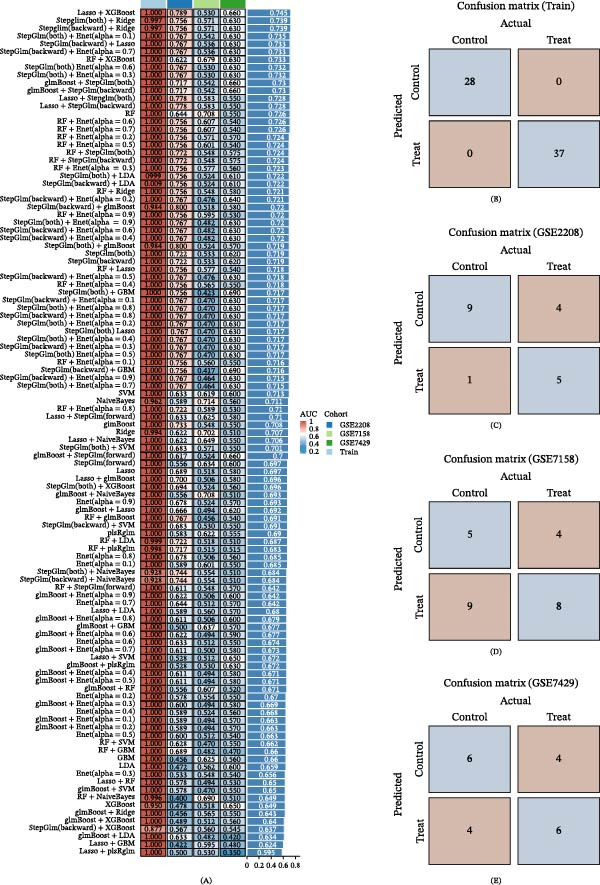
Identification of candidate targets based on 113 machine learning models. (A) Test results of the 113 machine learning models. (B–E) Confusion matrices for the training set and three validation sets.

To further screen for targets with strong associations between OP and LP, this study constructed 113 machine learning models based on the 127 potential targets to identify feature genes accurately. The results indicated that the Lasso+XGBoost model achieved the highest accuracy, evaluated using a linear scoring function and assessed by the AUC score (Figure 13A). Analysis of the confusion matrices for the training set and the three training sets demonstrated improved predictive performance for both the OP and LP groups (Figure 13B–E). Consequently, Lasso+XGBoost was identified as the optimal model, and the 24 genes associated with this model were selected as candidate targets for further investigation.

#### 3.4.4. Identification and Validation of Key Targets

The 24 candidate targets from the Lasso + XGBoost model were intersected with the 17 nodes from the MCODE network graph, ultimately yielding five targets—Amyloid Beta Precursor Protein (APP), Forkhead Box O1 (FOXO1), FOXO3, Jun Proto‐Oncogene (JUN), and Synuclein Alpha (SNCA)—identified as key targets through which LP influences OP (Figure 14A). The ROC curves for these key targets were validated in the training sets. Comprehensive analysis indicated that APP and FOXO1 possessed relatively higher diagnostic value, demonstrating superior predictive efficacy (Figure 14B). Furthermore, analysis of the expression levels of these key targets in the OP group versus the control group revealed that FOXO1 and FOXO3 were expressed at higher levels in the OP group. In contrast, the expression of APP, JUN, and SNCA was lower in the OP group compared to controls (Figure 14C). Notably, in the validation set, the performance of the aforementioned targets as independent biomarkers showed significant attenuation. Their expression levels did not demonstrate consistent or significant differences, and the AUC values were low, indicating limited discriminative ability (Supporting Information 4: Figures S1–S3). This finding diverges from the results obtained in our integrated dataset, though such discrepancy is not uncommon and precisely highlights the common challenges in biomarker development for complex diseases. We hypothesise that the potential reasons for this inconsistency include the following aspects. Firstly, there is inherent heterogeneity across datasets. The independent GEO cohorts exhibit substantial variation in patient population characteristics (such as ethnicity and age), disease stage, history of pharmacological treatment, and sample sources, which may dilute the specific signals identified in the relatively homogeneous discovery set. Secondly, despite rigorous batch normalisation applied to the discovery set in this study, residual technical variation between different platforms (such as distinct batches of microarray chips) may still affect the precise quantification of individual gene expression levels. Finally, and most plausibly, the pathological mechanism of OP is typically driven by subtle, coordinated changes across multiple molecular pathways rather than by the influence of a single gene. What our multivariate analysis captured in the training set is likely this synergistic effect. Consequently, when each target is evaluated individually in the validation set, the modest contribution of any single gene is insufficient to support significant diagnostic value. This interpretation is also consistent with the “multi‐target, multi‐pathway” mode of action revealed through our network pharmacology analysis [30].

Figure 14Analysis and evaluation of key targets. (A) Venn diagram of key targets. (B) ROC validation of the five key targets. (C) Differential expression of key targets between OP and control groups. (D) Correlation analysis among key targets.

**Figure:**
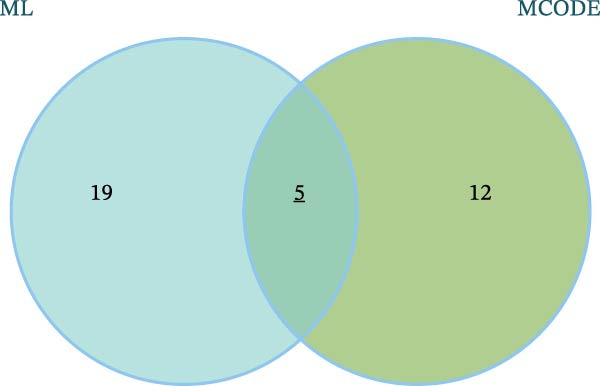
(A)

**Figure:**
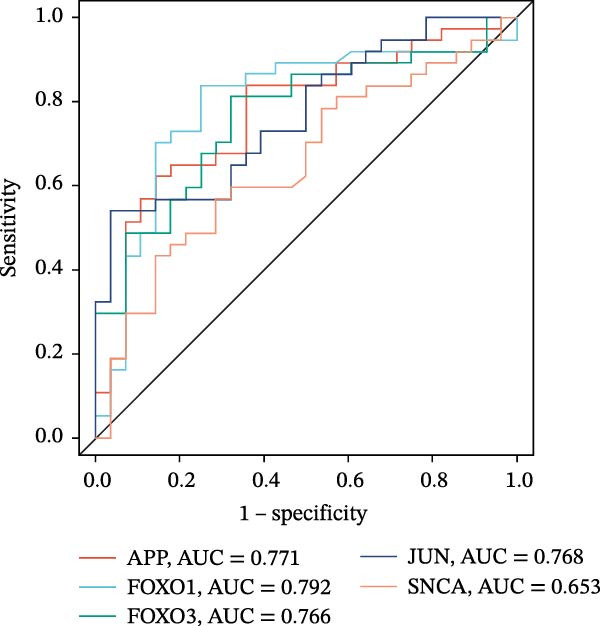
(B)

**Figure:**
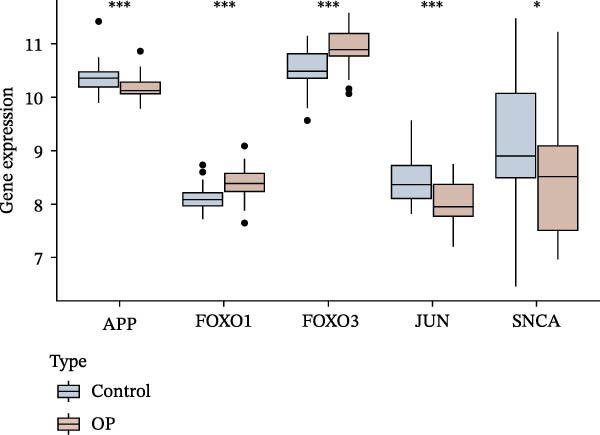
(C)

**Figure:**
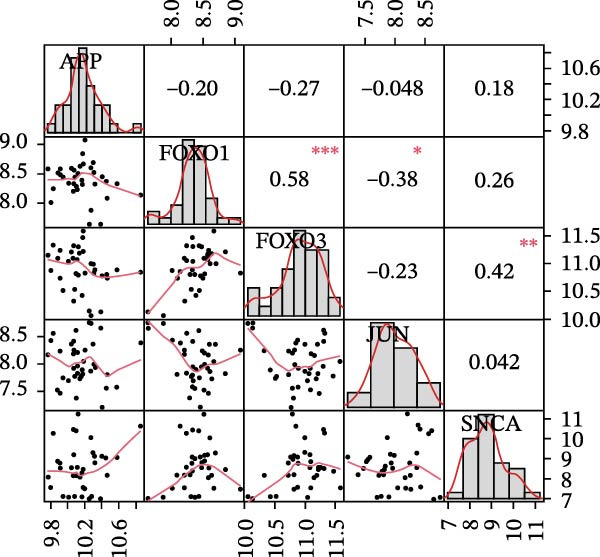
(D)

Finally, an analysis of the relationships between these genes showed a significant positive correlation between FOXO1 and FOXO3 (OR: 0.58, *p* < 0.001), a significant negative correlation between FOXO1 and JUN (OR: −0.38, *p* < 0.05), and a significant positive correlation between FOXO3 and SNCA (OR: 0.42, *p* < 0.01), as shown in Figure 14D.

## 4. Discussion

This study employed an integrated analytical strategy combining bibliometric and bioinformatics approaches to systematically elucidate the research landscape and molecular mechanisms in the OP and ATG field. The bibliometric analysis clearly delineated three evolutionary phases in this domain: the initial exploratory phase (2008–2017), characterised by slow annual growth in publications, marking the accumulation of fundamental theories and technologies (e.g., CRISPR‐Cas9, key molecular tools for ATG); the rapid development phase (starting in 2018), where annual publication numbers increased sharply and reached a sustained high plateau, reflecting the formation of field consensus and maturation of research paradigms, thereby establishing ATG’s role as a core regulator of bone metabolism; and the deepening and cross‐disciplinary phase (post‐2020), where research hotspots evolved from classical pathways like ’mTOR’ and ’mitophagy’ towards translational medicine directions such as ’stem cells’ and ’rheumatoid arthritis’, and most crucially, witnessed the emergence of ’LP’ as a robust new frontier. This evolutionary trajectory indicates that after solidifying the core mechanisms of ATG, the field is now committed to exploring more refined upstream and downstream regulatory events and their roles within complex pathological networks. Consequently, this study focused on the ’LP’ frontier, aiming to investigate its specific role in OP independent of ferroptosis. We successfully identified five key OP–LP targets (APP, FOXO1, FOXO3, JUN, SNCA), providing a new molecular perspective for understanding OP–LP interactions. Importantly, our bioinformatics findings extend beyond correlation, suggesting that LP may actively orchestrate the inflammatory and immune dysregulation prevalent in the osteoporotic bone microenvironment. This positions LP not merely as a passive biomarker of oxidative damage, but as a potential upstream driver that bridges metabolic stress with the chronic low‐grade inflammation central to OP pathogenesis.

Keyword co‐occurrence and burst analyses further revealed the evolution of research hotspots, with drug‐related clusters such as #1 (Denosumab) and #2 (Vitamin D) indicating a gradual shift in research focus towards clinical applications. The emergence of burst terms like ’Romosozumab’ and ’Teriparatide’ reflects the trend in drug development targeting ATG‐related pathways. This evolution demonstrates that the research paradigm in the OP–ATG field has transitioned from ’phenomenological description’ to ’mechanistic investigation’, from ’single processes’ to ’interconnected networks’, and from ’basic research’ to ’translational applications’. Early explorations of fundamental mechanisms such as ’oxidative stress’ and ’ER stress’ have progressively shifted towards research on pathways and targets such as ’mTOR’, ’mitophagy’, and ’RANKL’. In recent years, cross‐disease, multi‐mechanism directions such as ’LP’ and ’rheumatoid arthritis’ have emerged. Furthermore, our bibliometric analysis identified ’LP’ as a frontier keyword that has emerged within the last 3 years in OP and ATG research, suggesting its potential as an independent biological event in bone metabolism studies. However, current research predominantly situates LP within the ferroptosis framework, regarding it as a hallmark feature of ferroptosis. A series of studies has successfully established a ’LP‐ferroptosis‐bone cell dysfunction (ATG)‐OP’ cascade model [12, 16]. Therefore, this study innovatively positions LP as its core research subject, aiming to elucidate whether it possesses regulatory functions in OP that are independent of ferroptosis.

Relevant studies indicate that the disruption of mitochondrial integrity leads to dysregulation of oxidative phosphorylation, resulting in impaired ATP synthesis and exacerbated oxidative stress [31], which is considered a significant factor in the pathogenesis of various degenerative diseases, including OP. As a downstream event, LP is the terminal product of ROS attacking polyunsaturated fatty acids in biomembranes, generating various reactive aldehydes (such as MDA, 4‐HNE, and acrolein). These aldehydes subsequently propagate LP and play significant roles in its cytotoxic effects [32–34]. Furthermore, excessive ROS directly causes oxidative damage to cells and disrupts the function of both osteoblasts and osteoclasts [35]. A complex interplay exists between ATG and LP: moderate ATG can clear the toxic products generated by LP, promoting cell survival, whereas intense LP can trigger a blockage of autophagic flux or even more severe cell death programmes. While basal ATG alleviates oxidative stress by removing ROS‐generating components, excessive activation of lipophagy and ferritinophagy can promote ferroptosis by enhancing iron release and LP [36].

Therefore, rather than conventionally relegating it merely to the upstream events of ferroptosis, we innovatively considered LP as an independent and core BP. Using bioinformatics methods, we directly constructed the molecular connection between LP and OP to identify the regulatory targets and pathways linking them. Firstly, by integrating GEO datasets and LP‐related genes, we screened 127 common OP–LP targets. GO and KEGG enrichment analysis revealed that the interaction between OP and LP does not rely on a single pathway but is achieved through a complex, synergistic multi‐pathway network. The pathways within this network are interwoven, collectively shaping the pathological microenvironment of OP, with LP serving as a key intersecting node throughout.

Primarily, a robust inflammation and immune stress module is activated. This module includes the AGE‐RAGE signalling pathway in diabetic complications, the NF‐κB signalling pathway, the TNF signalling pathway, and NETs. The AGE‐RAGE signalling pathway drives inflammatory responses and oxidative stress [32–34], forming a positive feedback loop with classical inflammatory pathways, such as NF‐κB and TNF, thereby collectively amplifying inflammatory signals [37–40]. Crucially, the enrichment of these specific inflammatory and immune pathways is mechanistically significant. LP‐derived reactive aldehydes, particularly 4‐HNE, are established bioactive molecules that can act as agonists of the NF‐κB signalling pathway [41, 42]. Through protein modification and signalling activation, they promote the production of key inflammatory cytokines such as IL‐6 and TNF‐α, a link well‐established in chronic inflammatory conditions including bone and joint diseases [43]. Furthermore, the oxidative stress milieu fuelled by LP can activate innate immune cells, as oxidised lipid species can serve as DAMPs [44]. This activation includes neutrophils, potentially leading to the formation of NETs, a process directly driven by oxidative stress [45]. This provides a direct mechanistic bridge: LP products do not just correlate with but can initiate and perpetuate the very inflammatory responses that disrupt bone remodelling, creating a self‐amplifying loop of oxidative stress and inflammation. In parallel, widespread dysregulation of metabolism and energy homeostasis constitutes another major pillar. The attenuation of the oestrogen signalling pathway exacerbates oxidative stress and weakens osteoblast activity [28]; dysregulation of the PPAR signalling pathway and AMPK signalling pathway affects systemic and bone cell energy metabolism and fatty acid oxidation [46, 47]; meanwhile, the FoxO signalling pathway, as the core of cellular antioxidant defence, is activated under persistent stress to maintain homeostasis. However, its long‐term activation might also inhibit osteogenic differentiation, acting as a “double‐edged sword” [48–51]. Of particular importance, the enrichment of lipid metabolism pathways, such as linoleic acid metabolism, directly provides abundant reaction substrates for LP [52].

Ultimately, the cell fate decision hub becomes imbalanced under multiple forms of stress. The oxidative stress (represented by the chemical carcinogenesis–ROS pathway) and inflammatory microenvironment resulting from the aforementioned pathways directly activate the apoptosis pathway, inducing osteocyte apoptosis [28, 53]. To counter the primary source of ROS—dysfunctional mitochondria—the mitophagy pathway is activated in animals to perform quality control, a process potentially regulated by the HIF‐1 signalling pathway under hypoxic microenvironments [54]. However, in pathological states, the imbalance of these processes ultimately determines osteocyte death and functional decline collectively.

Throughout this network, LP acts as a common thread, tightly connecting pathways across all dimensions. It is both a downstream consequence of inflammatory pathways (e.g., AGE‐RAGE, TNF) and oxidative stress, and its products (e.g., MDA, 4‐HNE) can, in turn, act as signalling molecules to feedback‐activate pathways like NF‐κB, inhibit metabolic homeostasis, and disrupt the balance between ATG and apoptosis [55–57]. Therefore, the enrichment analysis results from this study indicate that LP serves as a core molecular bridge connecting multiple pathophysiological dimensions in OP, including inflammation, metabolic disorders, cell death, and ATG, rather than being an isolated phenomenon or merely an epiphenomenon subordinate to ferroptosis [58].

To precisely screen for the feature genes most closely associated with OP–LP, this study integrated 12 machine learning algorithms to construct 113 predictive models. Through a systematic evaluation of model robustness, we identified the optimal model. This was further combined with MCODE module analysis and ROC curve validation, ultimately pinpointing five key targets: APP, FOXO1, FOXO3, JUN, and SNCA. The successful identification of these targets not only provides a novel molecular perspective for revealing the regulatory mechanism of LP in OP that is independent of ferroptosis but also preliminarily sketches a potential OP–LP‐specific interaction network. We next delve into the specific roles of these targets within the context of bone cell biology.

Our study revealed a distinct differential expression pattern: FOXO1 and FOXO3 were significantly up‐regulated in the OP group, whereas APP, JUN, and SNCA were markedly down‐regulated. This pattern suggests they may undertake distinct or even antagonistic regulatory functions in the progression of OP.

The observed up‐regulation of FOXO1 and FOXO3 likely represents a compensatory cellular response to the heightened oxidative stress and LP environment in OP bone tissue. The FoxO transcription factors are pivotal defenders against oxidative stress in osteoblasts. They are known to transactivate genes encoding antioxidant enzymes, thus protecting osteoblasts from oxidative damage and thereby supporting bone formation [48–51]. However, the role of FoxOs in bone is a quintessential ‘double‐edged sword‘. Under conditions of persistent pathological stress, such as chronic oestrogen deficiency, sustained activation of FoxO signalling can inadvertently suppress osteoblastogenesis. This occurs through competitive interaction with key pro‐osteogenic pathways, most notably the Wnt/β‐catenin signalling cascade [49–60]. Therefore, the elevated expression of FOXO1/FOXO3 in our OP cohort might reflect a state of ‘compensatory overexpression but functional inhibition‘, where their protective role is overwhelmed, and their negative impact on bone formation becomes predominant, ultimately contributing to the osteogenic impairment in OP.

Conversely, the down‐regulation of JUN in the OP group points to a direct defect in the osteogenic transcriptional programme. As a core component of the AP‐1 transcription factor complex, c‐Jun is indispensable for osteoblast differentiation. Genetic studies have demonstrated that disruption of c‐Jun function severely impairs the expression of osteogenic marker genes and bone nodule formation [38, 61, 62]. The AP‐1 complex, often in collaboration with other transcription factors, regulates the cell fate decision of mesenchymal stromal cells toward the osteoblastic lineage. Thus, the low expression of JUN likely signifies a suppression of osteoblast differentiation capacity, directly linking the OP–LP microenvironment to a crippled bone formation machinery. Beyond its role in differentiation, JUN is a core component of the AP‐1 transcription factor complex, which is a classic mediator of cellular responses to inflammatory cytokines and oxidative stress [59, 63]. Its dysregulation in OP may thus reflect a broader disruption in the bone cell’s response to inflammatory cues within the LP‐rich microenvironment. Similarly, the interplay between upregulated FOXO factors and inflammatory pathways like NF‐κB can further skew the cellular fate toward a catabolic, pro‐inflammatory state, a role consistent with FOXO’s function as a key integrator of stress, metabolism, and immune signals in determining cellular outcomes [64]. This underscores that the identified key targets likely operate within a network where LP‐driven signals converge with inflammatory signalling to dictate osteoblast and osteoclast fate.

The downregulation of APP and SNCA provides intriguing cross‐disciplinary insights into bone metabolism. Beyond its role in Alzheimer’s disease, the APP and its proteolytic fragments have emerged as local regulators in bone. Specifically, the soluble fragment sAPPα, generated through non‐amyloidogenic cleavage of APP, has been shown to promote osteoblast proliferation and differentiation and enhance bone formation in vivo, potentially through interactions with the Wnt signalling pathway [65, 66]. Therefore, the reduced APP levels in OP may imply a loss of this intrinsic osteogenic support signal, exacerbating bone loss. Similarly, SNCA is best known for its role in Parkinson’s disease but is also expressed in bone cells. Its primary function is associated with the regulation of mitochondrial integrity and synaptic vesicle trafficking. In osteoblasts, proper mitochondrial function is critical for their high energy demands during matrix synthesis. Dysregulation of SNCA can lead to mitochondrial dysfunction, increased oxidative stress, and exacerbated apoptosis [67–69]. The low expression of SNCA in OP might indicate a failure in mitochondrial quality control within bone cells, further propelling the vicious cycle of oxidative damage and functional decline.

Correlation analysis among the key targets further revealed potential synergistic and antagonistic regulatory relationships within this network. The strong positive correlation between FOXO1 and FOXO3 suggests they might cooperatively function in countering oxidative stress within the OP–LP network. Conversely, the significant negative correlation between FOXO1 and JUN may reflect a critical dynamic balance within the osteoblast: under sustained LP stress, the upregulation of FoxOs might directly or indirectly suppress the pro‐differentiation activity of the JUN, representing a molecular trade‐off between stress defence and lineage‐specific function. The positive correlation between FOXO3 and SNCA hints at a possible co‐regulation in pathways related to mitochondrial stress response, warranting further investigation.

In summary, the five key targets identified in this study collectively form a complex regulatory framework for the OP–LP interaction network. The up‐regulation of FOXO1/3 reflects a stressed cellular defence mechanism that may paradoxically inhibit bone formation, while the down‐regulation of APP, JUN, and SNCA collectively contributes to impaired bone formation from different angles—namely, by diminishing osteogenic signals, crippling the core osteogenic transcription machinery, and disrupting mitochondrial homeostasis, respectively.

This study is the first to integrate bibliometric and bioinformatics methods, systematically revealing the interdisciplinary research trends and molecular mechanisms linking OP with ATG and LP. It successfully constructed multiple machine learning models to identify key targets, laying a crucial foundation for subsequent functional verification and drug development. However, several limitations should be acknowledged. Firstly, the data sources were confined to WOS and GEO; future studies could incorporate additional databases to enhance coverage. Secondly, the biological functions of the key targets have not been experimentally validated, necessitating further confirmation of their mechanisms of action through cellular or animal models. To bridge this gap, we propose a feasible experimental roadmap: initially, employing osteoblast cell lines to modulate key targets under LP inducers, assessing impacts on osteogenic differentiation and LP levels; subsequently, validating the most promising target in an OVX mouse model via AAV‐mediated gene therapy, with outcomes evaluated by micro‐CT and bone histomorphometry. This systematic validation will transition our computational findings toward biological and therapeutic relevance. Furthermore, although diverse machine learning models were employed, their generalisability requires strengthening through validation with external, independent datasets. Fourthly, it is important to note that this study did not perform stratified analyses for different subtypes of OP (e.g., PMO, age‐related/senile, or secondary OP). The bioinformatic analysis primarily utilised datasets from PMO patients, and the identified key targets and pathways may predominantly reflect the molecular characteristics of this specific subtype. Given that the aetiologies and pathophysiological mechanisms can vary significantly across different OP subtypes—such as the dominant role of oestrogen deficiency in PMO versus the multifaceted ageing processes in senile OP—the generalizability of our findings to all OP forms requires further investigation [70, 71]. Future research should aim to validate these key targets in cohorts specifically representing other OP subtypes to determine whether the role of LP is universal or subtype‐specific.

## 5. Conclusion

Beginning with a bibliometric analysis of the ‘OP‘ and ‘ATG‘ fields, this study has, for the first time, clearly delineated the full developmental panorama of the OP and ATG fields over the past two decades and accurately identified LP as a robust, emerging frontier within them. Building upon this, we utilised bioinformatics and multi‐model machine learning approaches to profoundly investigate the independent pathogenic significance of LP as a key biological event in OP. This led to the successful identification of five key targets—APP, FOXO1, FOXO3, JUN, and SNCA—with high diagnostic value. These findings not only validate the scientific nature of the bibliometric predictions but, more importantly, translate the macro‐level research trends into a concrete molecular regulatory network, providing a fundamentally new perspective for understanding the pathological mechanisms of OP. By elucidating how LP may actively drive inflammatory signalling and immune responses (e.g., via NF‐κB, NETosis) and identifying key molecular nodes (APP, FOXO1/3, JUN, SNCA) within this network, our study bridges the fields of redox biology and immunometabolism in bone, offering novel insights relevant to inflammatory aspects of OP and potential therapeutic strategies aimed at interrupting the LP‐inflammation axis.

## Author Contributions

Yu Zhou and Xin Li designed the study. Xin Li drafted the manuscript. Xin Li, Wei Dong, Yusong Liu, and Mengen Li collected the data and analysed the study. Yingtao Bai, Liqi Ng, Chunbao Wu, Yusong Liu, and Yu Zhou reviewed and edited the manuscript. Yu Zhou and Chunbao Wu contributed equally to this study.

## Funding

This study was supported by Chongqing Traditional Chinese Medical Scientific Research Project (Joint project of Chongqing Health Commission and Science and Technology Bureau) (Grant No. 2025ZYQN009) for financial support.

## Disclosure

All authors have read and approved the manuscript.

## Consent

The authors have nothing to report.

## Conflicts of Interest

The authors declare no conflicts of interest.

## Supporting Information

Additional supporting information can be found online in the Supporting Information section.

## Supporting information


**Supporting Information 1** Table S1: Top 10 keywords by frequency and centrality in OP–autophagy research.

**Supporting Information 2** Table S2: List of 127 overlapping targets between OP and LP.

**Supporting Information 3** Table S3: Complete results of GO and KEGG enrichment analysis for the 127 overlapping targets.

**Supporting Information 4** Figure S1: Validation analysis of the key targets in the verification dataset GSE2208. (A) ROC validation of the five key targets; (B) Differential expression of the key targets between the OP and control groups. Figure S2: Validation analysis of the key targets in the verification dataset GSE7158. (A) ROC validation of the five key targets; (B) Differential expression of the key targets between the OP and control groups. Figure S3: Validation analysis of the key targets in the verification dataset GSE7429. (A) ROC validation of the five key targets; (B) Differential expression of the key targets between the OP and control groups.

## Data Availability

The datasets used and/or analysed during the current study are available from the corresponding author upon reasonable request.
