# Supplementary material for: Uncovering the Independent Role of Lipid Peroxidation in Osteoporosis Through an Integrated Bibliometric and Bioinformatics Analysis
Source: Mediators Inflamm. 2026 Feb 16;2026:1064232. doi: 10.1155/mi/1064232 (PMC12909619; doi:10.1155/mi/1064232)
Supplement: Supplementary file 4 — Supporting Information 4 Figure S1: Validation analysis of the key targets in the verification dataset GSE2208. (A) ROC validation of the five key targets; (B) Differential expression of the key targets between the OP and control groups. Figure S2: Validation analysis of the key targets in the verification dataset GSE7158. (A) ROC validation of the five key targets; (B) Differential expression of the key targets between the OP and control groups. Figure S3: Validation analysis of the key targets in the verification dataset GSE7429. (A) ROC validation of the five key targets; (B) Differential expression of the key targets between the OP and control groups. [file MI-2026-1064232-s001.docx]

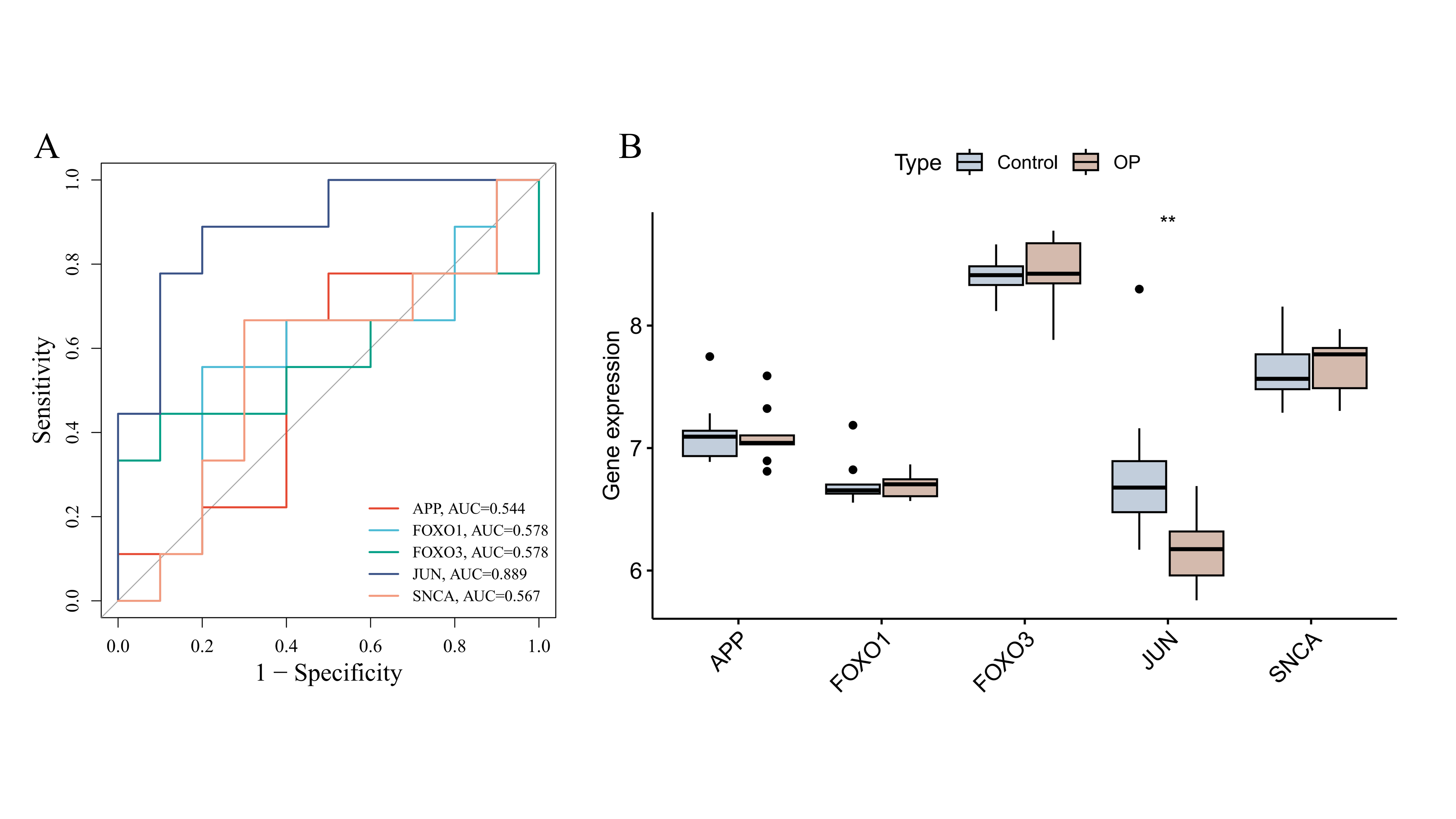


Supplementary Figure S1 Validation analysis of the key targets in the verification dataset GSE2208. A: ROC validation of the five key targets; B: Differential expression of the key targets between the OP and control groups.


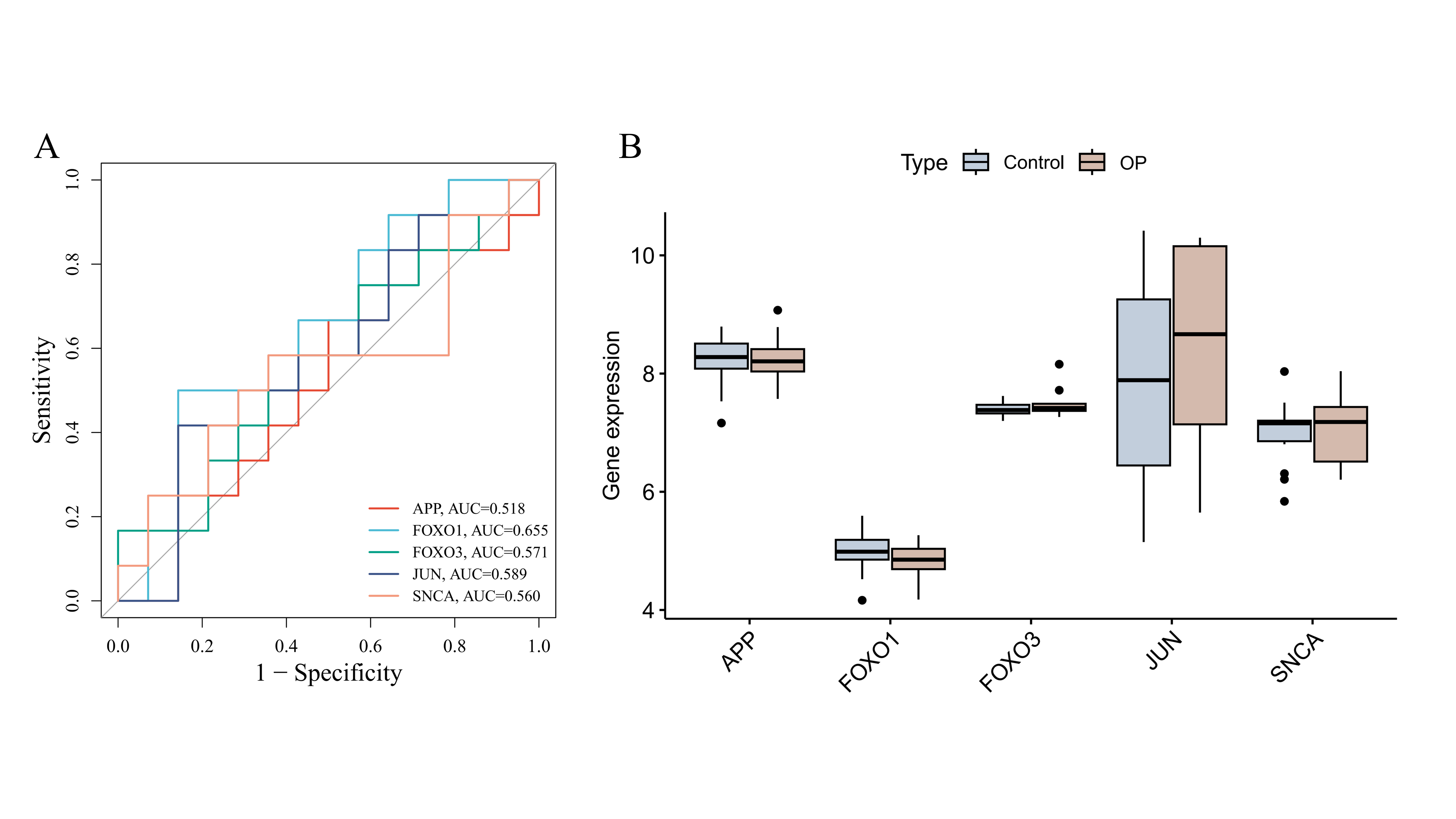


Supplementary Figure S2 Validation analysis of the key targets in the verification dataset GSE7158. A: ROC validation of the five key targets; B: Differential expression of the key targets between the OP and control groups.


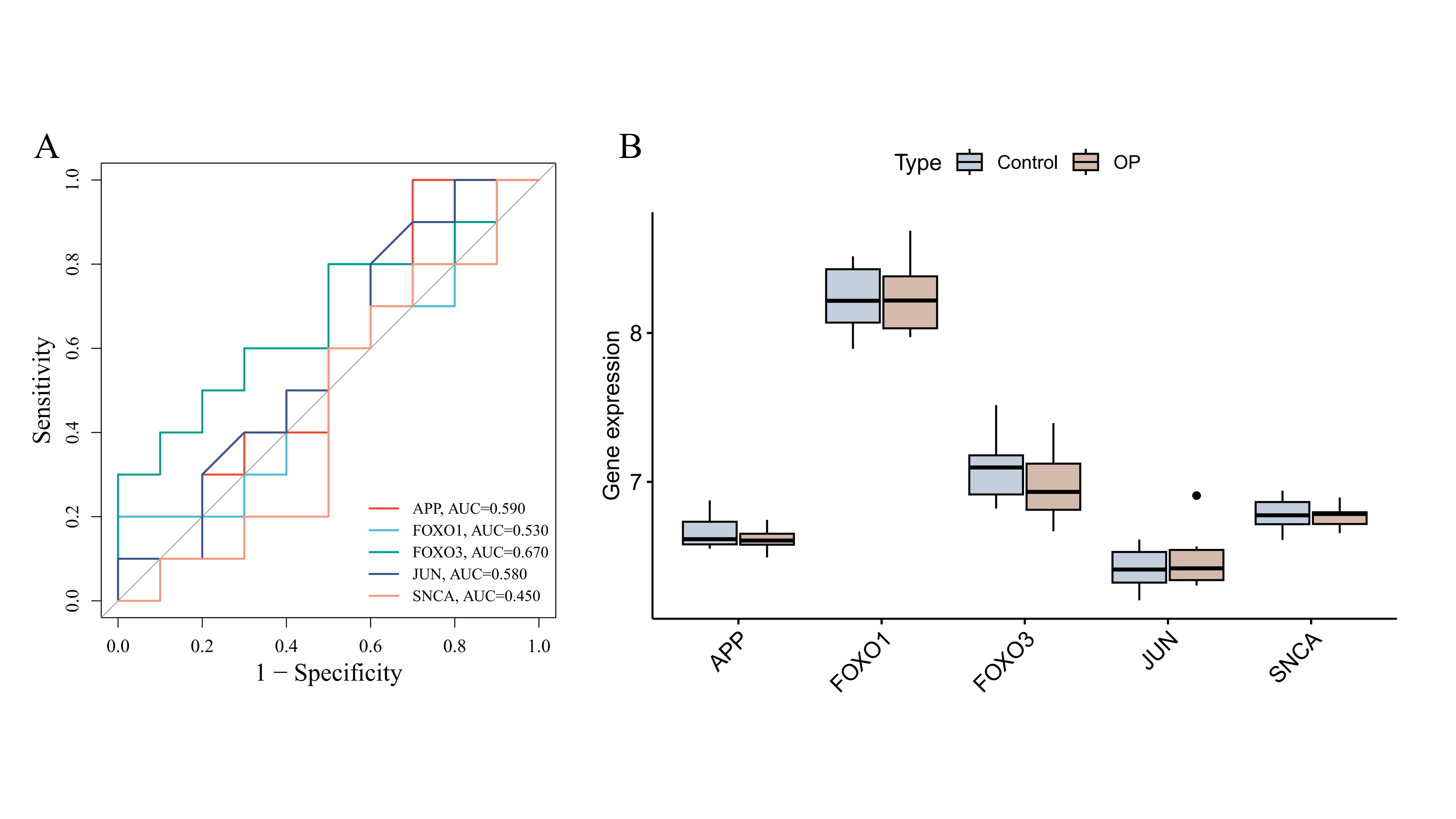


Supplementary Figure S3 Validation analysis of the key targets in the verification dataset GSE7429. A: ROC validation of the five key targets; B: Differential expression of the key targets between the OP and control groups.
